# Circular RNA hsa_circ_0000277 promotes tumor progression and DDP resistance in esophageal squamous cell carcinoma

**DOI:** 10.1186/s12885-022-09241-9

**Published:** 2022-03-04

**Authors:** Jiwei Cheng, Ruixiang Zhang, Ming Yan, Yin Li

**Affiliations:** Department of Thoracic Surgery, The Affiliated Cancer Hospital of Zhengzhou University, Henan Cancer Hospital, No.127 Dongming Road, Zhengzhou, 450008 Henan Province China

**Keywords:** hsa_circ_0000277, Esophageal squamous cell carcinoma, DDP resistance, miR-873-5p, SOX4

## Abstract

**Background:**

Circular RNAs (circRNAs) are well-known regulators of cancer progression and chemoresistance in various types of cancers. This study was performed to investigate the function of hsa_circ_0000277 in esophageal squamous cell carcinoma (ESCC).

**Methods:**

RNA levels were analyzed via the reverse transcription-quantitative polymerase chain reaction (RT-qPCR). Cell Counting Kit-8 (CCK-8) assay was applied to determine cell proliferation and half maximal inhibitory concentration (IC50) of cisplatin (DDP). Colony formation ability was evaluated by colony formation assay. Cell cycle and apoptosis were measured using flow cytometry. RNA immunoprecipitation (RIP), pull-down assay and dual-luciferase reporter assays were performed for target interaction analysis. The protein levels were determined through western blot. Xenograft models were established for researching hsa_circ_0000277 function in vivo.

**Results:**

Hsa_circ_0000277 expression was increased in ESCC cells and tissues, and it had important clinical significance. Downregulation of hsa_circ_0000277 repressed ESCC cell proliferation, colony formation, cell cycle, and DDP resistance. Hsa_circ_0000277 acted as a microRNA-873-5p (miR-873-5p) sponge and Sry-related high-mobility group box 4 (SOX4) was validated as a target of miR-873-5p. Moreover, hsa_circ_0000277/miR-873-5p axis and miR-873-5p/SOX4 axis regulated ESCC cell progression and DDP resistance. Hsa_circ_0000277/miR-873-5p axis activated SOX4/Wnt/β-catenin signaling pathway. *H*sa_circ_0000277 facilitated tumorigenesis and DDP resistance by miR-873-5p/SOX4 axis in vivo.

**Conclusion:**

These findings unraveled that hsa_circ_0000277 promoted ESCC progression and DDP resistance via miR-873-5p/SOX4/Wnt/β-catenin axis, showing a specific molecular mechanism of carcinogenesis and chemoresistance in ESCC.

**Supplementary Information:**

The online version contains supplementary material available at 10.1186/s12885-022-09241-9.

## Introduction

Esophageal squamous cell carcinoma (ESCC) is the most common histological subtype of esophageal cancers ranking as the seventh in incidence (572,000 new cases) and sixth in mortality (509,000 deaths) [1]. The risk factors of ESCC are numerous, such as smoking, alcohol consumption, gastroesophageal reflux disease, and obesity are [2]. Chemotherapy is an effective treatment for ESCC patients, but the chemoresistance leads to poor therapeutic effect and tumor recurrence [3]. It is important to study the molecular mechanism of chemoresistance in ESCC.

Circular RNAs (circRNAs) are specific noncoding RNAs (ncRNAs) derived from exons or introns by nonclassical back-splicing, and the covalent closed-loop structures endow high stability of circRNAs [4, 5]. The previous publications have highlighted the regulatory functions of exonic circRNAs in human cancers. For example, hsa_circ_403658 functioned as an oncogenic factor in bladder cancer [6] and hsa_circ_0007059 inhibited malignant progression of gastric cancer [7]. Hsa_circ_0000277 originates from Phosphodiesterase 3B (PDE3B) gene and exhibits significant upregulation in ESCC [8]. The biological role of hsa_circ_0000277 in ESCC is unknown.

MicroRNAs (miRNAs) are another regulatory class of ncRNAs in cancer development and drug resistance, including ESCC [9]. CircRNAs can serve as “miRNAs sponges” to suppress the miRNA binding to mRNAs, further affecting gene expression and cancer progression [10, 11]. Xu et al. concluded that hsa_circ_0031288/miR-139-3p/Bcl-6 axis regulated cervical cancer cell migration and invasion [12]. Circ-ABCB10 has been shown to enhance the resistance of paclitaxel in breast cancer via mediating Let-7a-5p/DUSP7 axis [13]. Liang et al. reported that miR-873 served as a tumor repressor in ESCC by targeting DEC2 [14]. Sry-related high-mobility group box 4 (SOX4) was proved to be a pro-cancer gene in ESCC [15]. The relation among hsa_circ_0000277, miR-873-5p, and SOX4 is not clear.

In addition, miR-129-5p suppressed proliferation and invasion of chondrosarcoma cells via targeting SOX4/Wnt/β-Catenin pathway [16] and miR-140-5p targeted SOX4 to retard tumorigenesis and progression in malignant melanoma by blocking the Wnt/β-Catenin pathway [17]. Thus, our final purpose is to disclose the hsa_circ_0000277/miR-873-5p/SOX4/Wnt/β-Catenin axis in cancer progression and chemoresistance of ESCC.

## Materials and methods

### Ethics and tissue specimens

All experiments strictly followed the Helsinki Declaration concerning the biomedical principles of human subjects, and all operating protocols were authorized by the Ethical Committee of Henan Cancer Hospital. Fifty-eight ESCC patients have signed the written informed consent forms. According to the clinicopathological analysis and follow-up visit, these patients were divided into different groups in tumor stage (I: *n* = 16; II: *n* = 27; III: *n* = 15), lymph node metastasis (LN-negative: *n* = 33; LN-positive: *n* = 25), and recurrence situation after cisplatin (DDP) therapy (non-recurrence: *n* = 22; recurrence: *n* = 36). Fifty-eight pairs of ESCC specimens and normal noncancerous samples (> 3 cm) were collected after the surgery at Henan Cancer Hospital. Tumor/normal > 1 was considered as hsa_circ_0000277 down-regulation (*n* = 8), and tumor/normal < 1 was considered as hsa_circ_0000277 up-regulation (*n* = 50). These tissues were snap-frozen in liquid nitrogen for 5 min and then stably saved in a − 80 °C ultra-low temperature freezer.

### Cell culture

Human esophageal epithelial cell line HET-1A and ESCC cell lines (EC9706 and KYSE30) were purchased from QCHENG BIO (Shanghai, China). Cell nutrient solution was prepared by Dulbecco’s modified eagle medium (DMEM; Gibco, Carlsbad, CA, USA), 10% fetal bovine serum (FBS; Gibco), 100 unit/mL penicillin, and 100 μg/mL streptomycin (Sigma-Aldrich, St. Louis, MO, USA). Then, cells were cultured in the humid environment with 5% CO_2_ at 37 °C. Cells were passaged by washing cells with phosphate buffer solution (PBS; Gibco) and digesting cells in trypsin (Gibco) for 2 min and then resuspending in the culture medium to subpackage at the ratio of 1:3.

### Cell transfection

Cells were sub-cultured in 6-well plates to reach 70% confluence. Then, short hairpin RNA (shRNA) lentivirus vectors (sh-circ_0000277#1, sh-circ_0000277#2 and sh-NC), miRNA mimics (miR-873-5p and miR-NC), miRNA inhibitors (anti-miR-873-5p and anti-NC), and pcDNA-SOX4/pcDNA vectors (SOX4 and pcDNA) were transfected using Lipofectamine™ 3000 Transfection Reagent (Invitrogen, Carlsbad, CA, USA). The above shRNA vectors and miRNAs were bought from GenePharma (Shanghai, China). In addition, pcDNA-SOX4 was constructed using the basic pcDNA vector (Invitrogen).

### RNA preparation and reverse transcription-quantitative polymerase chain reaction (RT-qPCR)

TRIzol™ Reagent (Invitrogen) was used for extraction of total RNA from tissues and cells. Nuclear and cytoplasmic RNA isolation was implemented by PARIS™ Kit (Invitrogen). The complementary DNA (cDNA) was synthesized by High-Capacity RNA-to-cDNA™ Kit (Applied Biosystems, Foster City, CA, USA), and the expression levels were quantified by TaqMan™ Fast Advanced Master Mix (Applied Biosystems) via the ABI7500 Fast Real-Time PCR System (Applied Biosystems). Glyceraldehyde-phosphate dehydrogenase (GAPDH) was used as an internal reference for circRNA and mRNAs, while small nuclear RNA U6 was exploited to normalize the levels of miRNAs. The relative expression levels were calculated by the comparative cycle threshold (2^−∆∆Ct^) method. Primers used for RT-qPCR analysis were listed in Table 1.

**Table 1:** Primer sequences used for RT-qPCR

Name	Primer sequences
Hsa_circ_0000277	Forward: 5′-TGGGATCGTAATAATGGCAAA-3′ Reverse: 5′-CTCCATTTCCACCTCCAGAA-3′
PDE3B	Forward: 5′-GGGAAGCGCCTCTTCATCCT-3′ Reverse: 5′-AAAGAATCATCTGTTCTCTG-3′
miR-136-5p	Forward: 5′-GCTGGGACTCCATTTGTTTT-3′ Reverse: 5′-CCAGTGCAGGGTCCGAGGT-3′
miR-1200	Forward: 5′-GCCGAGCTCCTGAGCCATTC-3′
	Reverse: 5′-CAGTGCAGGGTCCGAGGTAT-3′
miR-1294	Forward: 5′-TCGGCAGGTGTGAGGTTGGCAT-3′
	Reverse: 5′-CTCAACTGGTGTCGTGGA-3′
miR-421	Forward: 5′-GCCGAGATCAACAGACATTA-3′
	Reverse: 5′-CTCAACTGGTGTCGTGGA-3′
miR-517	Forward: 5′-TCGGCAGGCCTCTAGATGGAAG-3′
	Reverse: 5′-CAGTGCGTGTCGTGGAGT-3′
miR-873-5p	Forward: 5′-GCCGAGGCAGGAACTTGTGA-3′
	Reverse: 5′-GTGCAGGGTCCGAGGT-3′
SOX4	Forward: 5′-CAGCAAACCAACAATGCCGA-3′
	Reverse: 5′-GATCTGCGACCACACCATG-3′
ENAH	Forward: 5′-GTGGCTCAACTGGATTCAGCA-3′ Reverse: 5′-AGGAATGGCACAGTTTATCACGA-3′
GAPDH	Forward: 5′-ACAACTTTGGTATCGTGGAAGG-3′ Reverse: 5′-GCCATCACGCCACAGTTTC-3′
U6	Forward: 5′-GCTTCGGCAGCACATATACTAAAAT-3′ Reverse: 5′-CGCTTCACGAATTTGCGTGTCAT-3′

### Treatment of actinomycin D and ribonuclease R (RNase R)

Two milligrams per milliliter of actinomycin D (Millipore, Billerica, MA, USA) was added to the culture medium for 0 h, 6 h, 12 h, and 24 h. RNase R is an exoribonuclease exhibiting the 3′ to 5′ exonuclease activity to digest linear RNA species. Four micrograms of total RNA was digested by 2 μL RNase R (10 U/μL; Biovision, Milpitas, CA, USA) at 37 °C for 2 h, whereafter RT-qPCR was conducted to analyze the expression levels of hsa_circ_0000277 and PDE3B.

### Cell Counting Kit-8 (CCK-8) assay

Cell Counting Kit-8 (Sigma-Aldrich) was employed for cell proliferation detection. After transfection for 24 h, 48 h, or 72 h, cells were supplemented with CCK-8 solution with 10 μL/well. Four hours later, the absorbance at 450 nm was detected via the microplate reader. For determining the half maximal inhibitory concentration (IC50) of DDP, 2 × 10^4^ transfected cells were treated with DDP (Sigma-Aldrich) with the different concentrations (0 μM, 0.625 μM, 1.25 μM, 2.5 μM, 5 μM, 10 μM, 20 μM). DDP concentration at 50% cell viability was defined as the value of IC50.

### Colony formation assay

1 × 10^3^ transfected cells were transplanted into each well of the 6-well plates. After cell culture for approximate 10 days, the macroscopical colonies were fastened by 4% paraformaldehyde (Sigma-Aldrich) and dyed using 0.1% crystal violet (Sigma-Aldrich). The images of cloned plates were obtained, and the number was counted in each plate.

### Flow cytometry for cell cycle and apoptosis detection

Cell Cycle Assay Kit-PI/RNase Staining (Dojindo, Kumamoto, Japan) was used for cell cycle detection. The harvested cells were fixated in ice-cold 70% ethyl alcohol and stained with PI working solution, and then the cells were distinguished by a flow cytometer (BD Biosciences, San Diego, CA, USA) following the instruction book of the producer. FITC-Annexin V Apoptosis Detection Kit (BD Biosciences) was applied for apoptosis analysis. 1 × 10^5^ cells were stained with FITC-Annexin and PI following the users’ manual. The apoptotic cells were considered as cells at the early (Annexin+/PI−) and late (Annexin+/PI+) phases through the flow cytometer (BD Biosciences). The apoptotic cell percentage was calculated as below: apoptotic cells/total cells × 100%.

### RNA immunoprecipitation (RIP) assay

RIP experiment was performed using Imprint® RNA Immunoprecipitation Kit (Sigma-Aldrich). 1 × 10^6^ ESCC cells with the stable expression of sh-NC or sh-circ_0000277#1 were respectively lysed in RIP lysis buffer. Then, anti-Argonaute-2 (anti-Ago2) or anti-immunoglobulin G (anti-IgG) coated protein A magnetic beads were mixed with cell lysates and incubated at 4 °C overnight. After the treatment of proteinase K, the immunoprecipitated RNA was isolated for the examination of GAPDH and hsa_circ_0000277.

### Biotinylated RNA pull-down assay

C-1 magnetic beads (Life Technologies, Carlsbad, CA, USA) were incubated with hsa_circ_0000277 probe at room temperature for 2 h, using an oligo probe as the control probe. 1 × 10^7^ EC9706 and KYSE30 cells were added with the above probe-coupled beads at 4 °C overnight. The RNA complex was eluted from the magnetic beads, and the expression levels of miRNAs (miR-136-5p, miR-1200, miR-1294, miR-421, miR-517, and miR-873-5p) were assayed by RT-qPCR following the extraction of RNA by PureLink™ miRNA Isolation Kit (Invitrogen).

### Dual-luciferase reporter assay

By inserting the cDNA sequence into the pmirGLO luciferase vector (Promega, Madison, WI, USA), the wild-type (WT) and mutant-type (MUT) pmirGLO-control-hsa_circ_0000277 vectors (hsa_circ_0000277-WT and hsa_circ_0000277-MUT) were generated. Also, the pmirGLO-control vectors containing SOX4 3′UTR WT or MUT sequence (SOX4 3′UTR-WT,SOX4 3′UTR-MUT) were respectively constructed. EC9706 and KYSE30 cells were planted into the 24-well plates with 2 × 10^5^ cells/well. After co-transfection with vector (500 ng) and miR-873-5p or miR-NC (10 nM) for 2 days, the activities of firefly luciferase (FLUC) and *Renilla* luciferase (RLUC) were examined by the dual-luciferase reporter assay system (Promega). RLUC activity was used as the normalized control for FLUC. The fold-change of each luciferase plasmid was analyzed by the comparison of relative luciferase in miR-873-5p group with that in miR-NC group.

### Western blot

The extraction of total proteins and the determination of protein density were completed through RIPA buffer and BCA Protein Assay Kit. Then, western blot assay was performed with 40 μg proteins/sample, according to the operating procedures of previous reports [18, 19]. The used primary antibodies contained anti-cleaved-PARP (ab32064, 1:1000), anti-cleaved-caspase3 (ab2302, 1:1000), anti-SOX4 (ab90696, 1:1000), anti-β-catenin (ab6302, 1:1000), anti-c-myc (ab39688, 1:1000), anti-cyclin D1 (ab226977, 1:1000), and anti-GAPDH (ab9485, 1:2500). Goat Anti-Rabbit IgG H&L (HRP) second antibody (ab205718, 1:5000) was used to combine with primary antibodies, and then the conjugated signals were determined via the ECL Kit. All reagents and antibodies were purchased from Abcam (Cambridge, UK). The protein levels were quantified by the ImageLab software version 4.1 (Bio-Rad, Hercules, CA, USA). GAPDH was used as the housekeeping gene, and the fold-changes of proteins in experimental groups were calculated contrasted with the control groups.

### Tumor xenograft models and DDP sensitivity in vivo

In total, 20 male BALB/c nude mice (5-week-old, 22–24 g) were purchased from Shanghai Animal Experimental Center (Shanghai, China). Following the Guidelines for the Management and Use of Laboratory Animals of the NIH, mice were carefully reared in laminar flow cabinets without the specific pathogen in the Animal Laboratory department at The Affiliated Cancer Hospital of Zhengzhou University. All mice were firstly divided into two groups (*n* = 10 per group). 2 × 10^6^ EC9706 cells transfected with sh-circ_0000277#1 or sh-NC vector were subcutaneously injected into the right flanks of mice back. Tumor indicators (a: length, b: width) were measured by a digital caliper. When tumor volume (a × b^2^ × 0.5) reached 100–200 mm^3^, mice were divided into two sub-groups with *n* = 5.Then, mice were subjected to 2 mg/kg PBS or DDP treatment twice a week. Tumor volume was recorded every 7 days, and mice were sacrificed by displacing 30% air in the cabinets using the flow rate of CO_2_ after DDP or PBS injection for 28 days. RNA or protein extraction was performed from the excised tumors, and then RT-qPCR and western blot were applied to analyze the expression levels of hsa_circ_0000277, miR-873-5p, and SOX4. SOX4, β-catenin, c-myc, and cyclin D1 protein levels were measured by immunohistochemistry (IHC) assay [20]. This animal assay was ratified by the Animal Review Ethical Committee of Henan Cancer Hospital.

### Statistical analysis

All samples were determined in triplicate and all experiments were independently carried out for three times. SPSS 24.0 and GraphPad Prism 7 were used for statistical analysis. The experimental results were presented as the mean ± standard deviation (SD). The survival curve was generated and analyzed via Kaplan-Meier plot and log-rank test. Linear relations were analyzed by Pearson’s correlation coefficient in clinical samples. Student’s *t*-test and one-way analysis of variance (ANOVA) followed by Tukey’s test were used to compare the difference of groups. *P <* 0.05 indicated a significant difference.

## Results

### Hsa_circ_0000277 was upregulated in ESCC cells and its characteristics as a circRNA

**Fig. 1:**
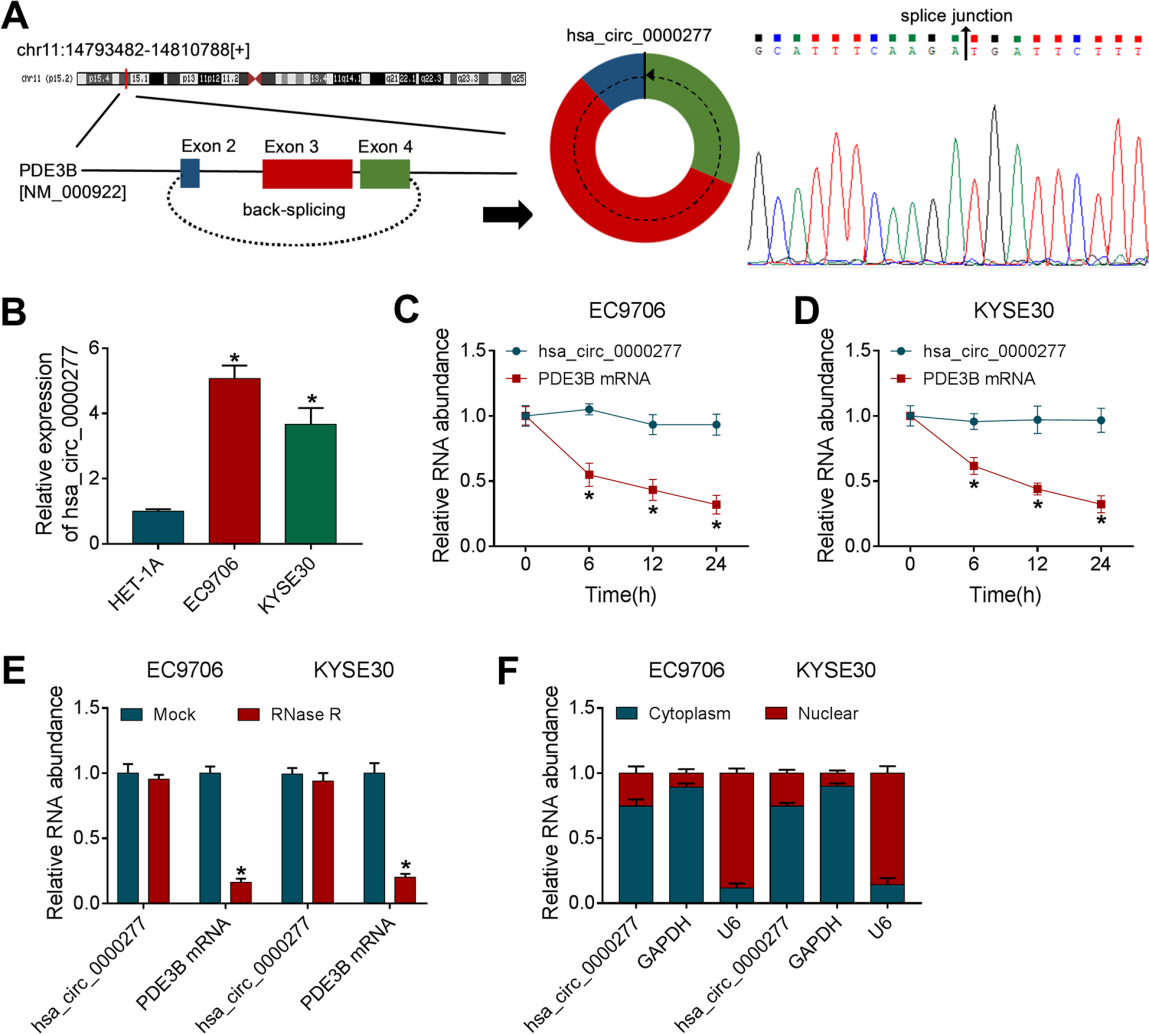
Hsa_circ_0000277 was upregulated in ESCC cells and its characteristics as a circRNA. **A** The back-splicing information of hsa_circ_0000277 and Sanger sequencing in the splice junction. **B** The detection of hsa_circ_0000277 was administrated exploiting RT-qPCR in ESCC (EC9706 and KYSE30) cells and control HET-1A cells. **C**, **D** The levels of hsa_circ_0000277 and its linear form (PDE3B mRNA) were assayed by RT-qPCR in EC9706 and KYSE30 cells treated with actinomycin D. **E** The RT-qPCR was used for analyzing hsa_circ_0000277 and PDE3B mRNA after RNA was exposed to RNase R. **F** Expression levels of hsa_circ_0000277, GAPDH and U6 were measured using RT-qPCR in the cytoplasmic or nuclear fraction. *P < 0.05

Hsa_circ_0000277 is a backing-splicing circular product derived from the exon 2-4 of PDE3B gene and its splice junction was confirmed by Sanger sequencing (Fig. 1A). RT-qPCR revealed that hsa_circ_0000277 was upregulated with more than 2-fold changes in EC9706 and KYSE30 cells contrasted with HET-1A cells (Fig. 1B). After treatment of actinomycin D, PDE3B mRNA level was quickly decreased 70% while hsa_circ_0000277 was almost unchanged at 24 h (Fig. 1C, D). AHsa_circ_0000277 was more resistant to exonucleolytic activity of RNase R than PDE3B mRNA (Fig. 1E). Additionally, hsa_circ_0000277 was mainly enriched in the cytoplasm of EC9706 and KYSE30 cells using GAPDH and U6 as control groups (Fig. 1F). Preliminarily, we affirmed that hsa_circ_0000277 was an upregulated circRNA in ESCC cells.

### Hsa_circ_0000277 was overexpressed in ESCC tissues and its clinical significance

**Fig. 2:**
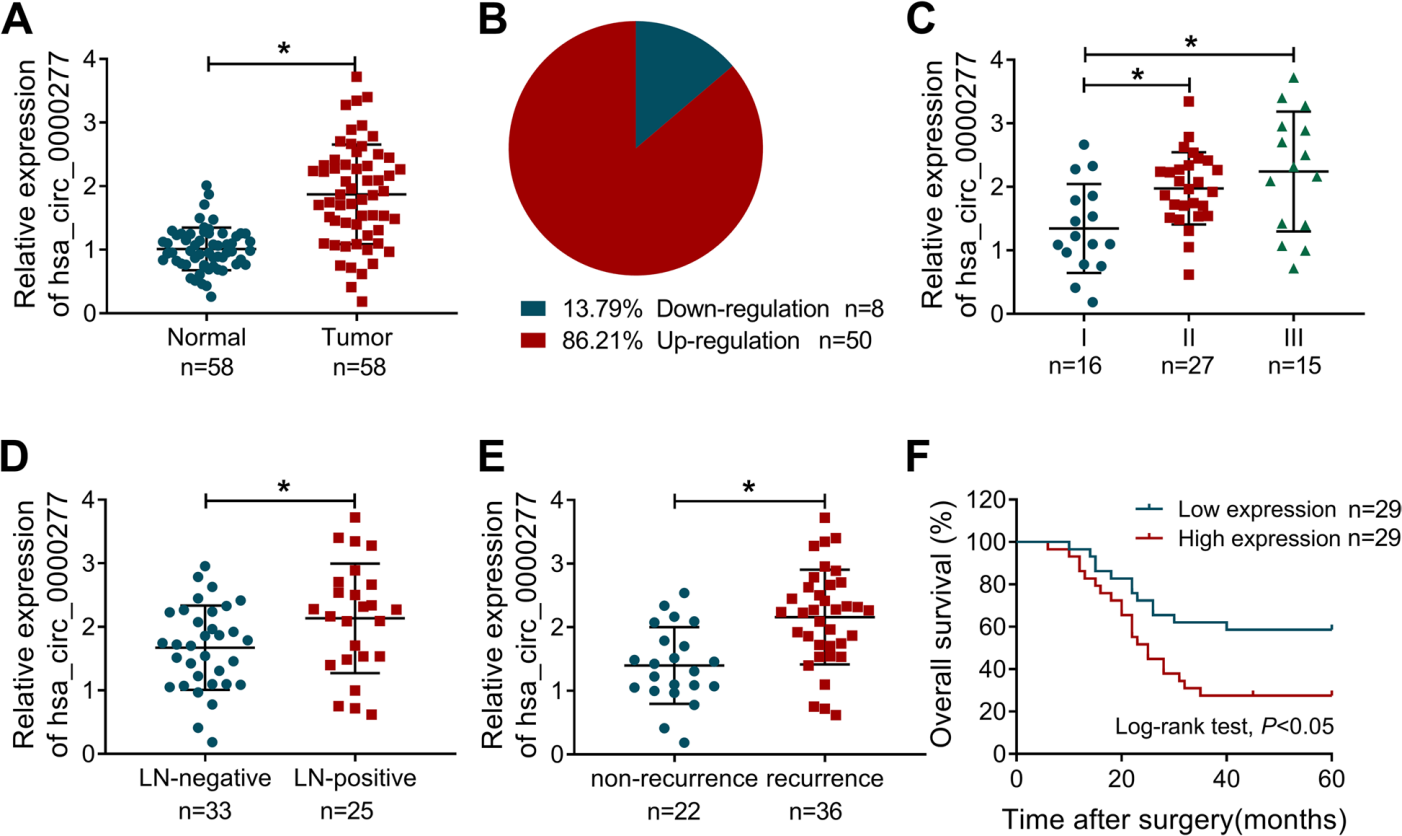
Hsa_circ_0000277 was overexpressed in ESCC tissues and its clinical significance. **A**, **B** The determination of hsa_circ_0000277 by RT-qPCR (**A**) and its expression distribution (**B**) in ESCC samples. **C**–**E** The RT-qPCR was performed to examine the hsa_circ_0000277 level in tumor stage tissues (**C**), LN-negative/ positive tissues (**D**), and non-recurrent/recurrent tissues (**E**). **F** Log-rank test was carried out to analyze the overall survival in ESCC patients expressed high or low hsa_circ_0000277. *P < 0.05

Then, we determined the hsa_circ_0000277 level in collected 58 paired tissues. Hsa_circ_0000277 was upregulated in 86.21% ESCC tissues (*n* = 50) while downregulated in 13.79% (*n* = 8) ESCC tissues, contrasted with normal non-cancerous tissues (Fig. 2A, B). In addition, our RT-qPCR analysis demonstrated that the upregulation of hsa_circ_0000277 was closely associated with tumor stage (Fig. 2C) and metastasis (Fig. 2D). The high expression of hsa_circ_0000277 was also detected in recurrent samples after DDP chemotherapy, implying that hsa_circ_0000277 was related to DDP resistance (Fig. 2E). Fifty-eight patients were divided into high and low expression groups according to the median value of hsa_circ_0000277 in ESCC tissues. The 5-year survival analysis after surgery indicated that overall survival was higher in ESCC patients with high hsa_circ_0000277 than that in those patients with low hsa_circ_0000277 (Fig. 2F). Also, clinical factors exhibited that high level of hsa_circ_0000277 was related to tumor growth, metastasis, and recurrence of ESCC patients (Table 2). Hence, hsa_circ_0000277 might be associated with tumor progression, metastasis, chemoresistance and poor prognosis in ESCC.

**Table 2:** Correlation of has_circ_0000277 expression with clinicopathologic features in ESCC patients

Parameters	*N* = 58	has_circ_0000277		*p*-value
		High *N* = 29	Low *N* = 29	
Age, years				
< 60	25	12	13	0.847
≥ 60	33	17	16	
Tumor size				
< 4	37	14	23	0.014
≥ 4	21	15	6	
TNM stage				
I–II	43	19	24	0.134
III	15	10	5	
Lymph node metastasis				
No	33	13	20	0.063
Yes	25	16	9	
DDP therapy				
Non-recurrence	22	5	17	0.001
Recurrence	36	24	12	

### Knockdown of hsa_circ_0000277 suppressed cell proliferation, colony formation, cell cycle, and DDP resistance in ESCC cells

**Fig. 3:**
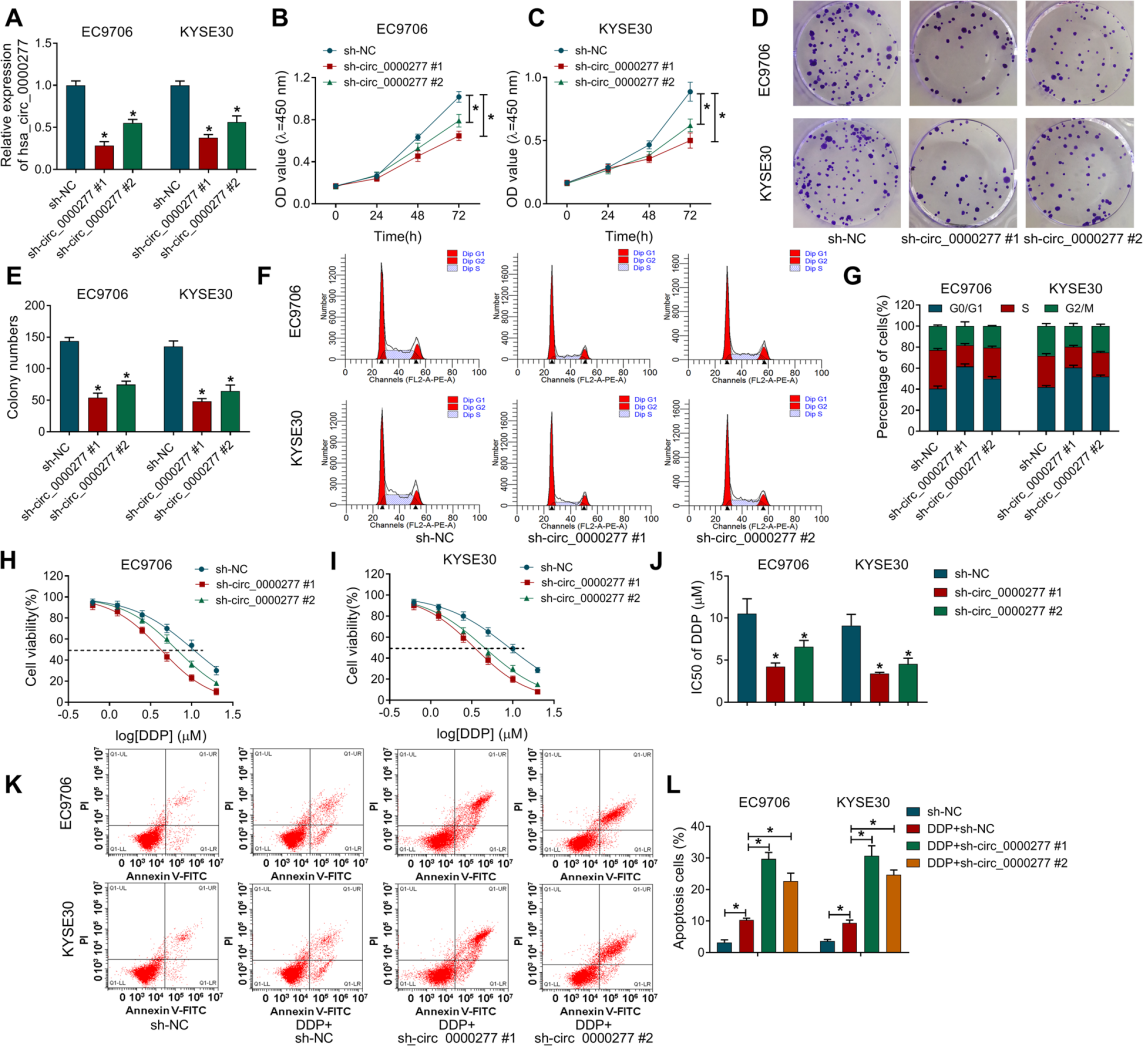
Knockdown of hsa_circ_0000277 suppressed cell proliferation, colony formation, cell cycle, and DDP resistance in ESCC cells. **A** Hsa_circ_0000277 was detected through RT-qPCR in EC9706 and KYSE30 cells with transfection of shRNA vectors (sh-NC, sh-circ_0000277#1 or sh-circ_0000277#2). **B**–**G** Cellular proliferation (**B**–**C**), colony formation (**D**–**E**), and cell cycle (**F**–**G**) were respectively assessed by CCK-8, colony formation assay and flow cytometry. **H**–**J** IC50 of DDP was assayed by CCK-8. **K**–**L** The apoptotic effect of hsa_circ_0000277 downregulation on ESCC was analyzed by flow cytometry under DDP treatment. *P < 0.05

Hsa_circ_0000277 expression was knocked down by shRNA vectors in EC9706 and KYSE30 cells, and hsa_circ_0000277#1 exhibited better effectiveness than hsa_circ_0000277#2 (Fig. 3A). The inhibition of cell proliferation (Fig. 3B, C) and colony formation ability (Fig. 3D, E) suggested that silence of hsa_circ_0000277 impeded cell growth. Also, transfection of sh-circ_0000277#1 or sh-circ_0000277#2 vector blocked the transition of cells from G0/G1 to S phase to inhibit the progression of cell cycle (Fig. 3F, G). To research the effect of hsa_circ_0000277 on chemoresistance, cell viability was measured by CCK-8 assay under the condition of DDP treatment. The analysis of cell viability (Fig. 3H, I) demonstrated that IC50 value of DDP was reduced after the knockdown of hsa_circ_0000277 (Fig. 3J). Moreover, flow cytometry manifested that knockdown of hsa_circ_0000277 aggravated the DDP-induced cell apoptosis in EC9706 and KYSE30 cells to inhibit DDP resistance (Fig. 3K, L). Western blot further showed that circ_0000277 downregulation upregulated the levels of pro-apoptotic cleaved-PARP and cleaved-caspase3 in DDP-treated EC9706 and KYSE30 cells (Supplementary Fig. 1A-B). In addition, EdU assay indicated that hsa_circ_0000277 downregulation repressed cell proliferation and flow cytometry demonstrated that cell apoptosis was promoted with only inhibition of hsa_circ_0000277 in EC9706 and KYSE30 cells (Supplementary Fig. 2A-B). Taken together, ESCC progression and DDP resistance were inhibited by knocking down hsa_circ_0000277.

### Hsa_circ_0000277 acted as a sponge for miR-873-5p in ESCC cells

**Fig. 4:**
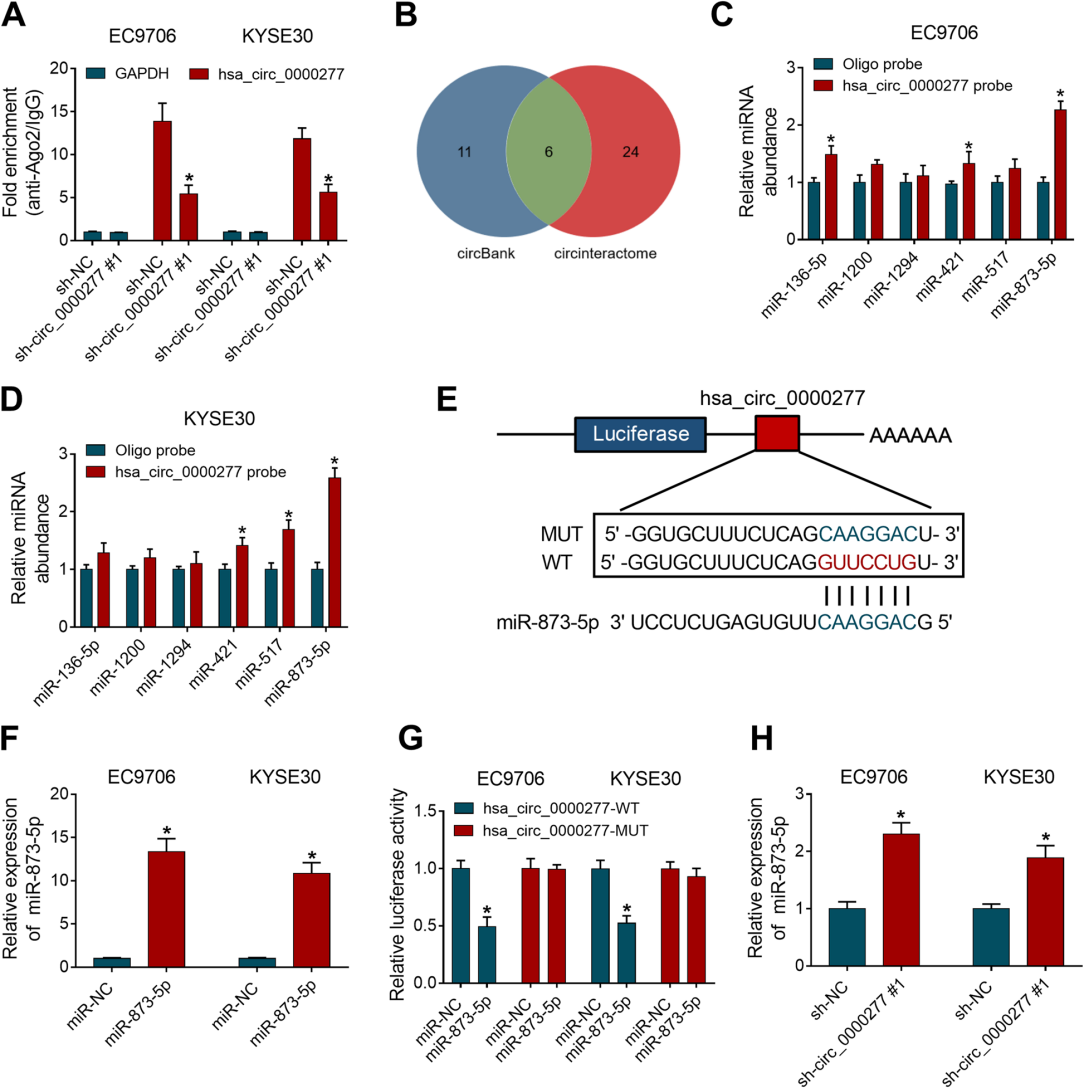
Hsa_circ_0000277 acted as a sponge for miR-873-5p in ESCC cells. **A** RIP assay was administrated to study whether hsa_circ_0000277 could bind to Ago2 protein. **B** Venn Diagram analysis was used for selecting the common miRNAs from circBank and circinteractome. **C**, **D** Pull-down assay with biotinylated hsa_circ_0000277 probe was implemented to explore which miRNA was captured by hsa_circ_0000277. **E** The binding sites between hsa_circ_0000277 and miR-873-5p in circinteractome and the presentation of the mutated sites. **F** The overexpression effect of miR-873-5p mimic on miR-873-5p was evaluated by RT-qPCR. **G** Dual-luciferase reporter assay was performed for affirming that hsa_circ_0000277 could interact with miR-873-5p. **H** The RT-qPCR was employed to determine miR-873-5p level after transfection of sh-NC or sh-circ_0000277#1. *P < 0.05

Ago2 protein can act on miRNAs and drive RNA-induced silencing complex (RISC) to affect RNA expression in the upstream or downstream of miRNAs [21]. RIP data suggested that hsa_circ_0000277 was captured by Ago2 contrasted to GAPDH and the enrichment of hsa_circ_0000277 was lower in sh-circ_0000277#1 group than that in sh-NC group, implicating that hsa_circ_0000277 might bind to Ago2 protein (Fig. 4A). CircBank and circinteractome were used to seek the miRNAs targets combined with hsa_circ_0000277. Venn Diagram analysis displayed that 6 miRNAs (miR-136-5p, miR-1200, miR-1294, miR-421, miR-517 and miR-873-5p) were common in both two software (Fig. 4B). RNA pull-down assay with biotinylated hsa_circ_0000277 probe indicated that miR-421 and miR-873-5p were abundantly pulled down by hsa_circ_0000277 probe contrasted with oligo probe in EC9706 and KYSE30 cells (Fig. 4C, D). Then, miR-873-5p with significant capture by hsa_circ_0000277 was selected for subsequent experiment. The binding sites between hsa_circ_0000277 and miR-873-5p in circinteractome were shown in Fig. 4E. The miR-873-5p overexpression was induced by miR-873-5p mimic relative to miR-NC group (Fig. 4F). Dual-luciferase reporter assay showed that miR-873-5p upregulation decreased 50% relative luciferase activity of hsa_circ_0000277-WT plasmid, but it had no significant difference of luciferase activity in hsa_circ_0000277-MUT plasmid (Fig. 4G). The miR-873-5p level was upregulated in sh-circ_0000277#1-transfected ESCC cells, compared with sh-NC-transfected cells (Fig. 4H). All these results revealed that hsa_circ_0000277 acted as a sponge for miR-873-5p.

### Hsa_circ_0000277 targeted miR-873-5p to regulate tumor process and DDP resistance in ESCC cells

**Fig. 5:**
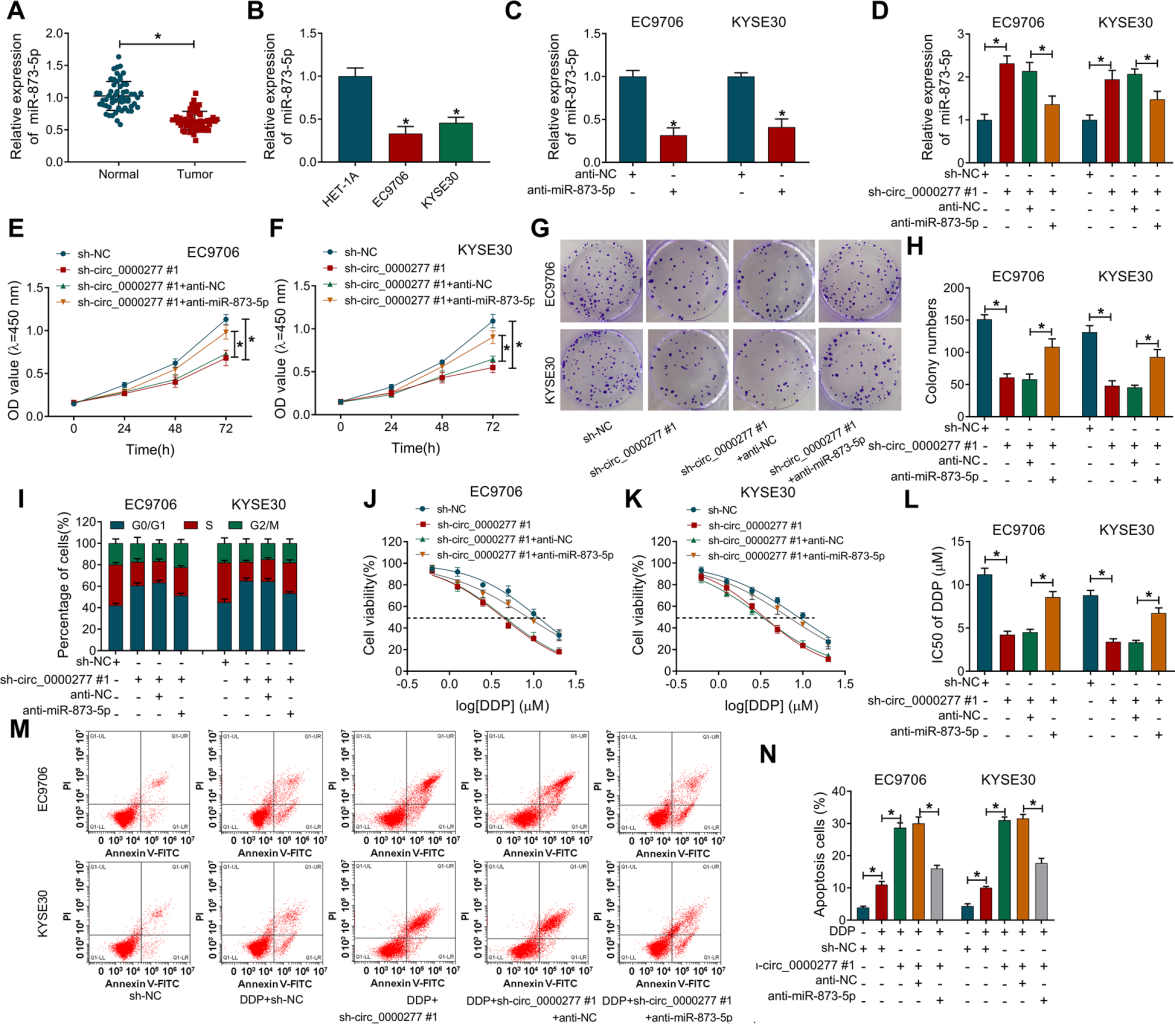
Hsa_circ_0000277 targeted miR-873-5p to regulate tumor process and DDP resistance in ESCC cells. **A**, **B** The relative level of miR-873-5p was detected via RT-qPCR in ESCC tissues (**A**) and cells (**B**). **C** Transfection efficiency of anti-miR-873-5p was assessed by RT-qPCR. **D** The miR-873-5p quantification was performed by RT-qPCR after EC9706 and KYSE30 cells were transfected with sh-NC, sh-circ_0000277#1, sh-circ_0000277#1+anti-NC, or sh-circ_0000277#1+anti-miR-873-5p. **E**–**I** Tumor progression was analyzed using cell proliferation by CCK-8 (**E**, **F**), clonal ability by colony formation assay (**G**, **H**), and cell cycle by flow cytometry (**I**) in four transfection groups. **J**–**N** CCK-8 and flow cytometry were respectively used to measure the IC50 of DDP (**J**–**L**) and cell apoptosis (**M**, **N**) after DDP treatment and the above transfection. *P < 0.05

The level of miR-873-5p was downregulated in ESCC tissues and cells (EC9706 and KYSE30) contrasted with normal tissues and HET-1A cell line (Fig. 5A, B). RT-qPCR showed that transfection efficiency of anti-miR-873-5p was significant in EC9706 and KYSE30 cells (Fig. 5C), and anti-miR-873-5p reversed sh-circ_0000277#1-mediated miR-873-5p upregulation (Fig. 5D). The sh-circ_0000277#1-induced suppression of cell proliferation (Fig. 5E-F), colony formation (Fig. 5G, H) and cell cycle (Fig. 5I) was counteracted by the introduction of anti-miR-873-5p. IC50 detection (Fig. 5J–L) and apoptosis analysis (Fig. 5M, N) revealed that DDP resistance inhibition caused by hsa_circ_0000277 downregulation was also partly restored after the miR-873-5p level was reduced. Meanwhile, miR-873-5p inhibitor eliminated the promoting effects of sh-circ_0000277#1 on the cleaved-PARP and cleaved-caspase3 protein levels after DDP treatment (Supplementary Fig. 1C-D). All in all, hsa_circ_0000277 regulated ESCC progression and DDP resistance by targeting miR-873-5p.

### SOX4 was a downstream gene for miR-873-5p in ESCC cells

**Fig. 6:**
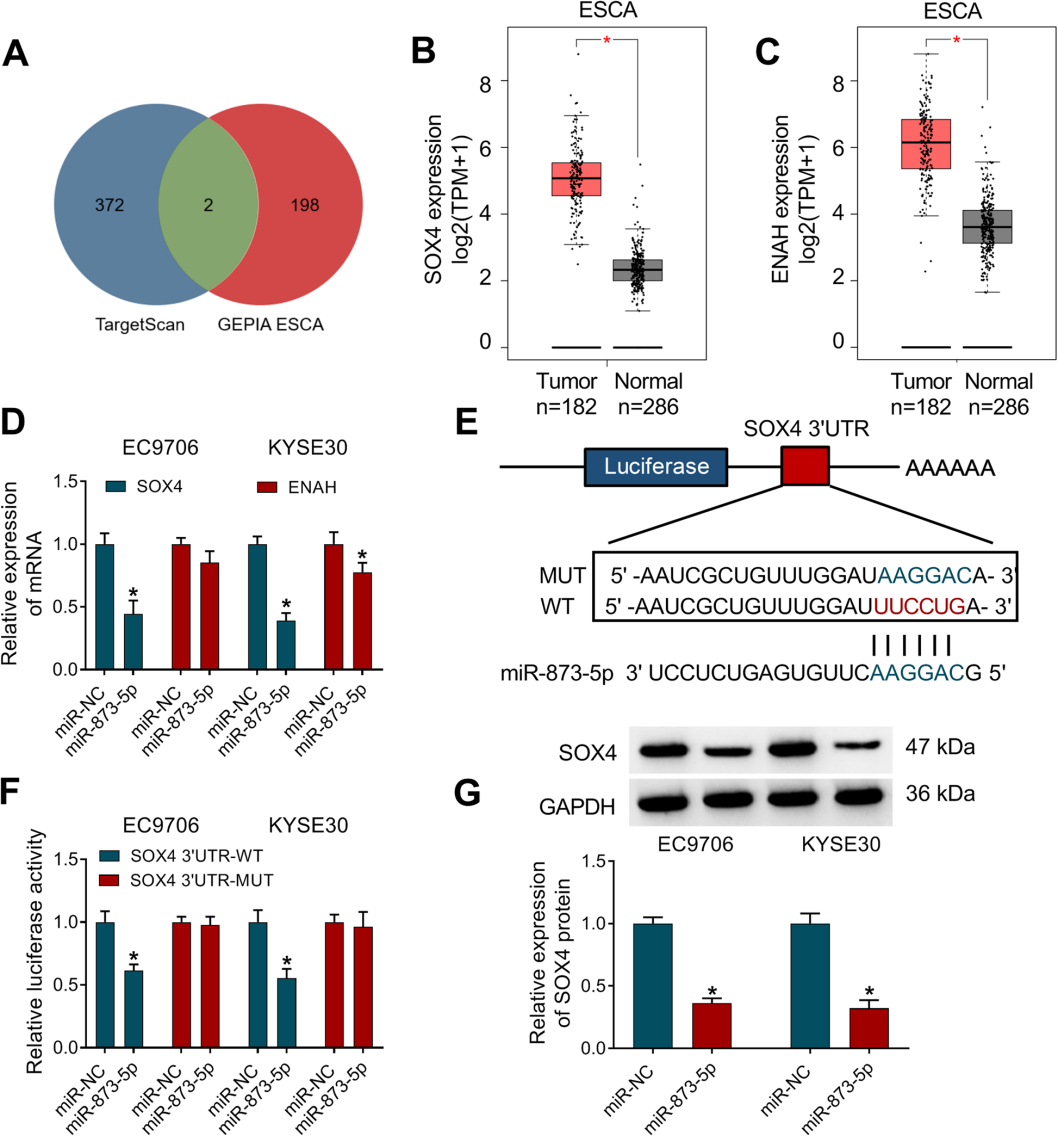
SOX4 was a downstream gene for miR-873-5p in ESCC cells. **A** The common target genes in TargetScan and GEPIA ESCA were sought by Venn Diagram analysis. **B**, **C** GEPIA database showed the expression of SOX4 (**B**) and ENAH (**C**) in ESCA samples. **D** SOX4 and ENAH mRNA levels were detected by RT-qPCR in EC9706 and KYSE30 cells transfected with miR-NC or miR-873-5p. **E** The combined target sites of SOX4 3′UTR for miR-873-5p were shown by TargetScan. **F** The measurement of luciferase activity was performed by the dual-luciferase reporter system after co-transfection of SOX4 3′UTR-WT or SOX4 3′UTR-MUT and miR-873-5p or miR-NC. **G** SOX4 protein expression was examined applying with western blot in miR-NC or miR-873-5p transfection group. *P < 0.05

The Targetscan software was used to search the target genes for miR-873-5p in the downstream, and 374 genes were predicted to have binding sites of miR-873-5p. After the comparison with the TOP200 upregulated genes of esophageal carcinoma (ESCA) in GEPIA database (http://gepia.cancer-pku.cn/detail.php), only two genes (SOX4 and ENAH) were chosen as the candidate target genes for miR-873-5p (Fig. 6A). As Fig. 6B, C depicted, SOX4 and ENAH were upregulated by approximate 3-fold changes in ESCA samples. After overexpression of miR-873-5p, SOX4 mRNA level was repressed with more significant difference than ENAH in EC9706 and KYSE30 cells (Fig. 6). Thus, SOX4 was used for the following target analysis of miR-873-5p. According to the binding sites of SOX4 3′UTR for miR-873-5p, point mutation was performed for studying the interaction between SOX4 and miR-873-5p (Fig. 6E). Upregulation of miR-873-5p led to the luciferase inhibition only in SOX4 3′UTR-WT plasmid of EC9706 and KYSE30 cells (Fig. 6F). Western blot showed that miR-873-5p mimic reduced the protein expression of SOX4 compared to miR-NC transfection (Fig. 6G). Therefore, miR-873-5p directly targeted SOX4 in ESCC cells.

### MiR-873-5p was identified as an inhibitor in ESCC development and DDP resistance by downregulating SOX4

**Fig. 7:**
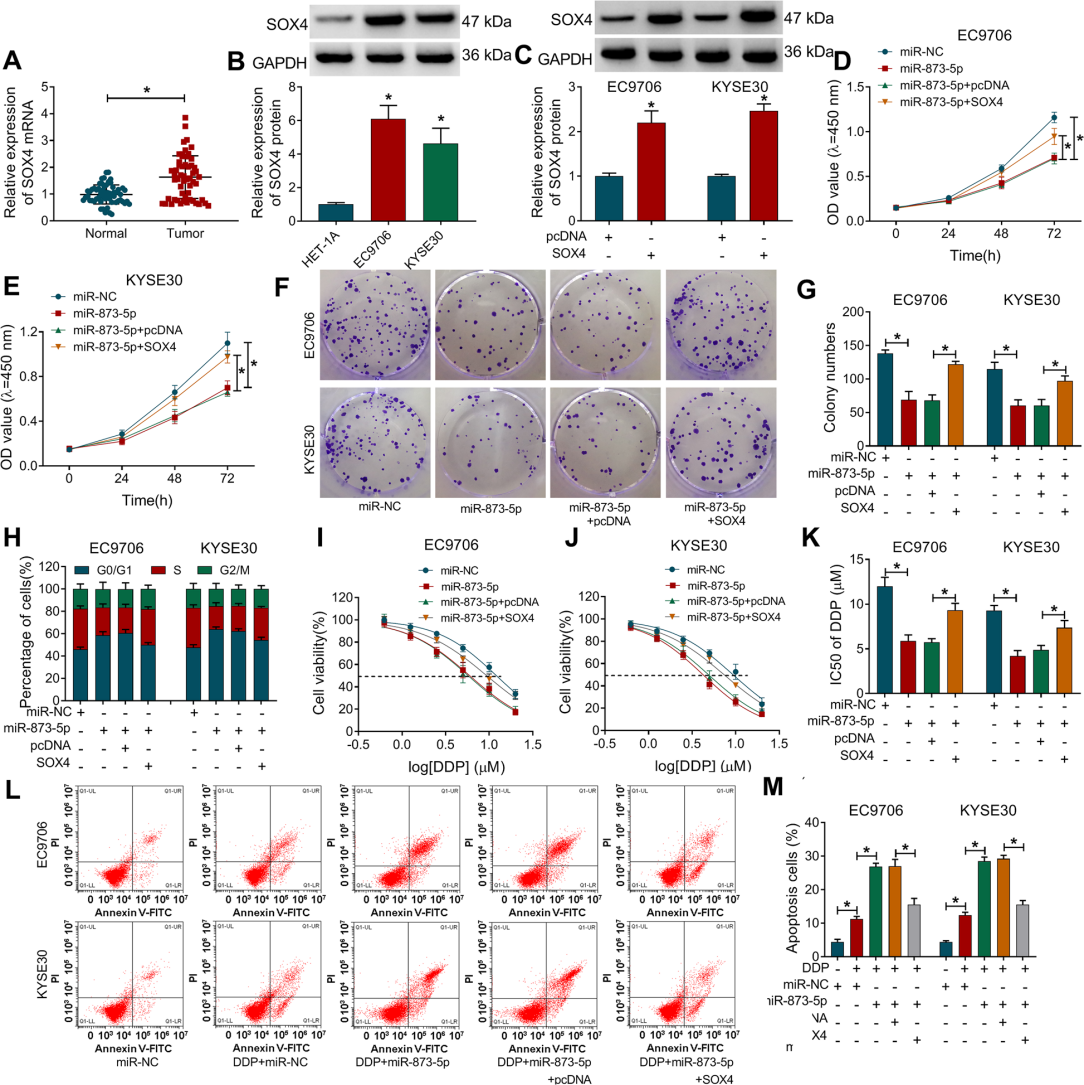
MiR-873-5p was identified as an inhibitor in ESCC development and DDP resistance by downregulating SOX4. **A**, **B** The RT-qPCR and western blot were conducted for the analysis of SOX4 mRNA level in ESCC tissues (**A**) and protein level in ESCC cells (**B**). **C** After transfection of pcDNA or SOX4 vector, the protein expression of SOX4 was determined via western blot. **D**–**H** ESCC progression was evaluated by examining cell proliferation via CCK-8 (**D**, **E**), colony formation capacity via colony formation assay (**F**, **G**), and cell cycle progression via flow cytometry (**H**) following transfection of miR-NC, miR-873-5p, miR-873-5p+pcDNA, or miR-873-5p+SOX4. **I**–**M** After the above transfection under the treatment of DDP, IC50 of DDP (**I**–**K**) and cell apoptosis rate (L–**M**) were respectively examined via CCK-8 and flow cytometry. *P < 0.05

SOX4 mRNA expression was elevated in ESCC samples relative to normal samples (Fig. 7A). Also, protein level of SOX4 was higher in EC9706 and KYSE30 cells than that in HET-1A cells (Fig. 7B). SOX4 vector was constructed for overexpressing SOX4, and western blot indicated that transfection efficiency of SOX4 was great (Fig. 7C). These repressive effects of miR-873-5p on cellular processes including cell proliferation (Fig. 7D, E), clonal capacity (Fig. 7F, G), and cell cycle (Fig. 7H) were abrogated by the introduction of SOX4. Transfection of miR-873-5p reduced the IC50 of DDP (Fig. 7I–K) and exacerbated cell apoptosis (Fig. 7L, M) after DDP treatment in EC9706 and KYSE30 cells, whereas these effects were recovered following the upregulation of SOX4. The miR-873-5p-mediated upregulation of cleaved-PARP or cleaved-caspase3 protein expression was also attenuated after SOX4 overexpression in DDP-treated cells (Supplementary Fig. 1E-F). Thus, miR-873-5p served as a repressor in cancer development and DDP resistance in ESCC cells by targeting SOX4.

### Hsa_circ_0000277/miR-873-5p axis activated the SOX4/Wnt/β-catenin signaling pathway

**Fig. 8:**
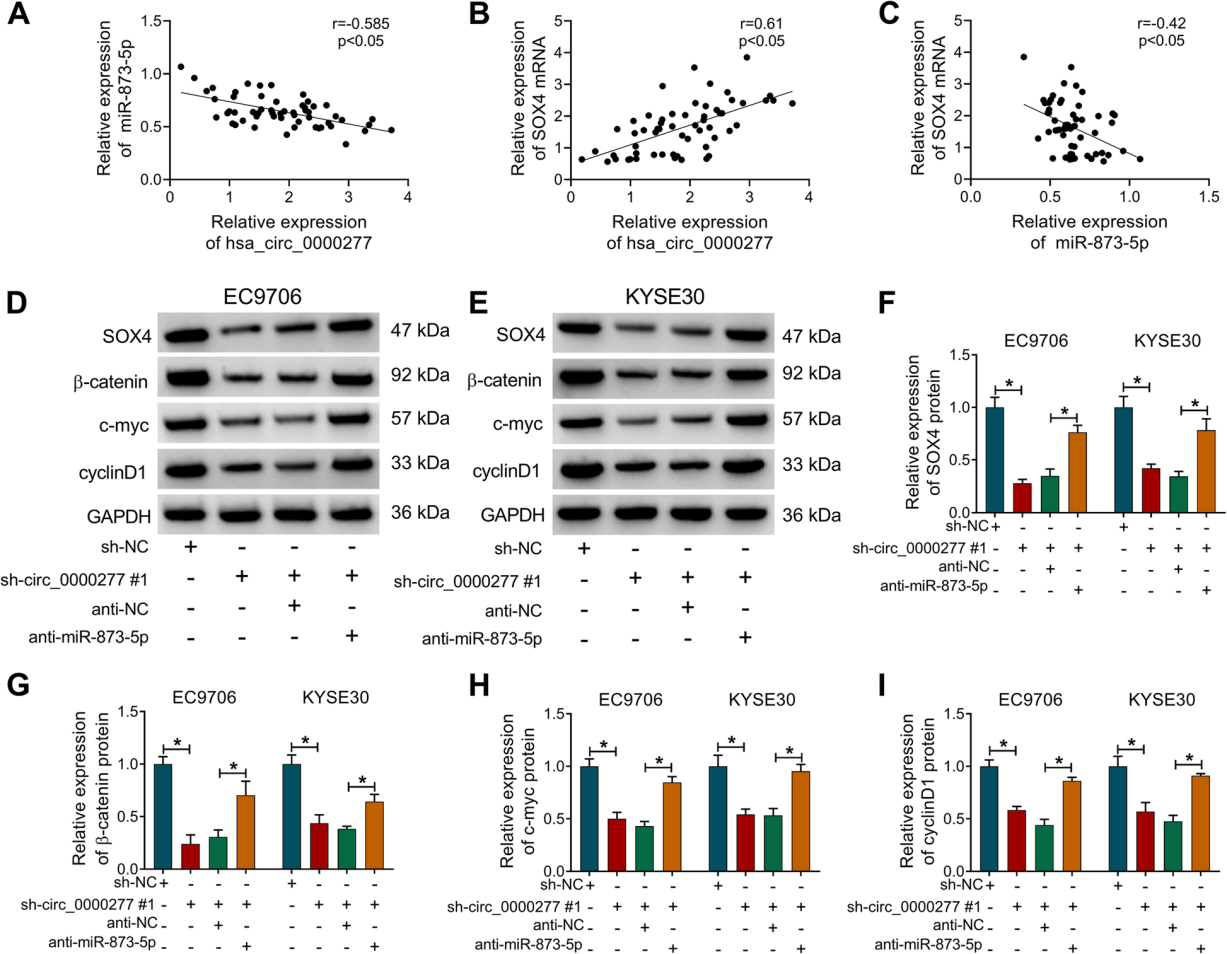
Hsa_circ_0000277/miR-873-5p axis activated the SOX4/Wnt/β-catenin signaling pathway. **A**–**C** Pearson’s correlation coefficient was used to analyze the linear relations of hsa_circ_0000277/miR-873-5p (**A**), hsa_circ_0000277/SOX4 (**B**), and miR-874-5p/SOX4 (**C**) in ESCC samples. **D**–**I** Western blot was performed for the protein detection of SOX4, β-catenin, c-myc, and cyclin D1 in the sh-NC, sh-circ_0000277#1, sh-circ_0000277#1+anti-NC, or sh-circ_0000277#1+anti-miR-873-5p group. *P < 0.05

Pearson’s correlation coefficient was used to analyze the association among hsa_circ_0000277, miR-873-5p, and SOX4 in clinical ESCC samples. The expression of hsa_circ_0000277 was negatively associated with miR-873-5p level (*r* = − 0.585, *p* < 0.05) (Fig. 8A), while hsa_circ_0000277 was positively related to SOX4 (*r* = 0.61, *p* < 0.05) (Fig. 8B). In addition, there was also a negative relation (*r* = − 0.42, *p* < 0.05) between miR-873-5p and SOX4 levels (Fig. 8C). Furthermore, the effects of hsa_circ_0000277/miR-873-5p on SOX4 and Wnt/β-catenin signaling pathway were analyzed. The protein results (Fig. 8D, E) demonstrated that hsa_circ_0000277 downregulation prominently decreased the protein levels of SOX4 (Fig. 8F) and Wnt/β-catenin-related proteins (β-catenin, c-myc, and cyclin D1) (Fig. 8G–I), while anti-miR-873-5p transfection mitigated these effects. The above evidence clarified that hsa_circ_0000277 promoted the SOX4 expression to activate the Wnt/β-catenin signaling pathway via targeting miR-873-5p.

### Hsa_circ_0000277 targeted miR-873-5p/SOX4 to facilitate tumorigenesis and DDP resistance in vivo

**Fig. 9:**
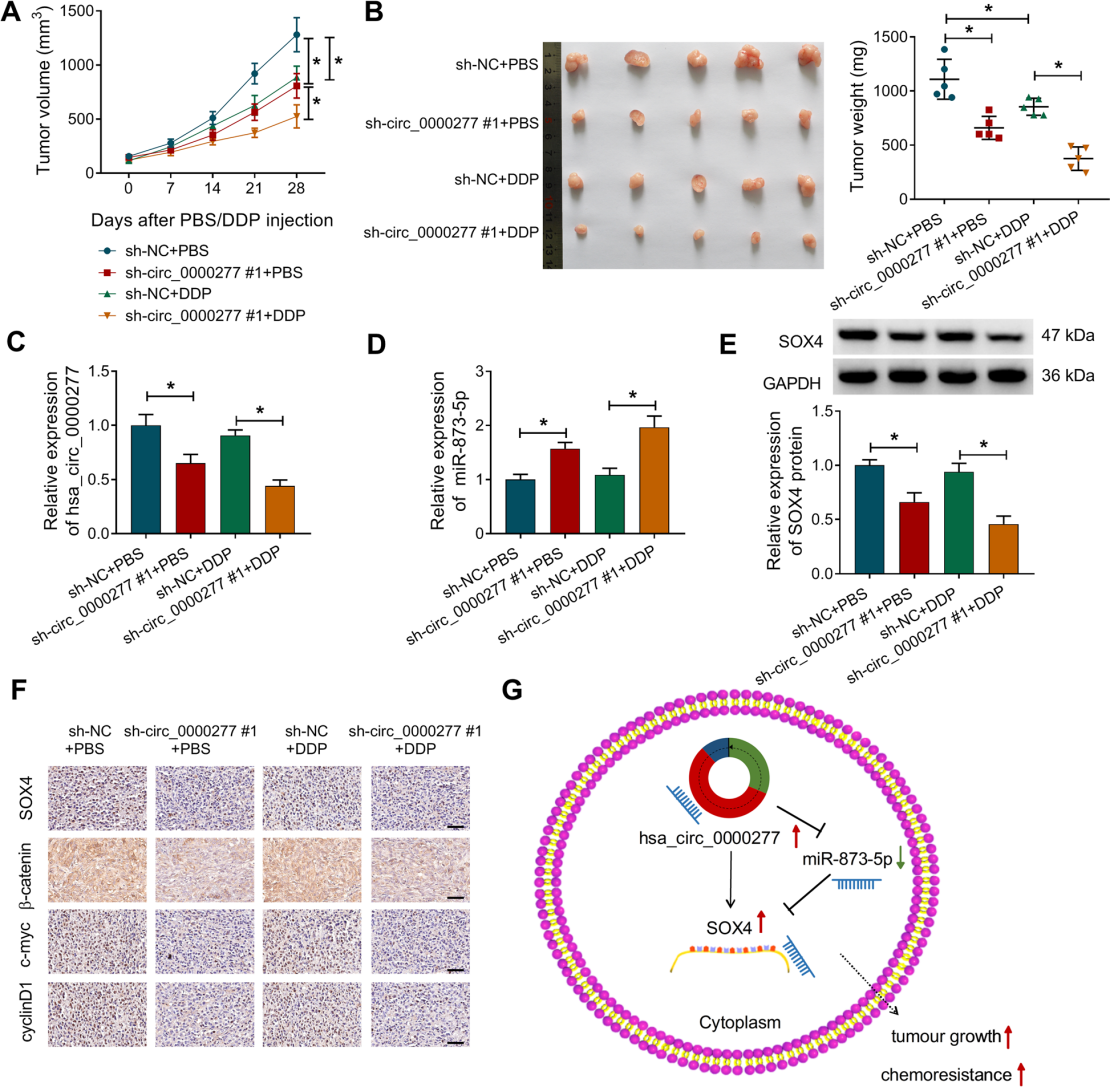
Hsa_circ_0000277 targeted miR-873-5p/SOX4 to facilitate tumorigenesis and DDP resistance in vivo. **A** Tumor volume was measured in each group every 7 days after PBS/DDP injection. **B** After 28 days, mice were sacrificed and tumor weight was determined. **C**, **D** RT-qPCR was used for the level detection of hsa_circ_0000277 (**C**) and miR-873-5p (**D**). **E** SOX4 protein level was assayed in each group using western blot. **F** SOX4, β-catenin, c-myc, and cyclin D1 protein levels in tissues were examined by IHC assay. **G** Schematic illustration of the hsa_circ_0000277/miR-873-5p/SOX4 axis in tumor growth and chemoresistance of ESCC in vivo. *P < 0.05

To research the role of hsa_circ_0000277 in vivo, xenograft models were established by injecting the sh-circ_0000277#1 or sh-NC-transfected EC9706 cells and PBS/DDP. Tumor volume (14–28 days) and weight (at 28 days) were found to be reduced in sh-circ_0000277#1+PBS and sh-NC+DDP groups (by comparison with sh-NC+PBS) as well as sh-circ_0000277#1+DDP group (in contrast to sh-NC+DDP) (Fig. 9A, B). Thus, hsa_circ_0000277 knockdown or DDP inhibited tumor growth and hsa_circ_0000277 downregulation relieved DDP resistance to promote DDP-induced tumorigenesis inhibition. The expression analysis of hsa_circ_0000277 (Fig. 9C), miR-873-5p (Fig. 9D), and SOX4 (Fig. 9E) in sh-circ_0000277#1 groups contrasted with sh-NC groups proved the hsa_circ_0000277/miR-873-5p/SOX4 axis in vivo. IHC assay also indicated that hsa_circ_0000277 induced inhibitory effects on SOX4, β-catenin, c-myc and cyclin D1 protein levels in tumor tissues (Fig. 9F). In addition, knockdown of hsa_circ_0000277 downregulated the protein levels of cleaved-PARP and cleaved-caspase3 in tumor tissues with PBS or DDP treatment (Supplementary Fig. 1G-H). As the schematic illustration in Fig. 9G, hsa_circ_0000277 promoted tumor growth and chemoresistance by targeting miR-873-5p to upregulate the expression of SOX4.

## Discussion

Dysregulated circRNAs have played oncogenic or inhibitory roles in cancers of the digestive system [22]. In the present study, we identified that hsa_circ_0000277 functioned as a tumor promoter to accelerate the biological development of ESCC and DDP resistance by depending on the miR-873-5p/SOX4/Wnt/β-catenin signaling pathway.

Through the characteristic analysis in ESCC cells, we found that hsa_circ_0000277 was more stable than its linear inform, and it was mainly localized in the cytoplasm. As for its clinical significance, the upregulated hsa_circ_0000277 was closely associated with tumor stage, lymph node metastasis and recurrence post-chemotherapy in ESCC patients. Additionally, hsa_circ_0000277 had crucial prognostic value for predicting poor survival. All these analyses manifested that hsa_circ_0000277 might be involved in the regulation of cancer progression and chemoresistance formation in ESCC.

Recent studies have suggested that circRNAs are key regulators in ESCC progression. CircFNDC3B has been considered as a tumorigenic factor to promote cell proliferation and migration in ESCC [23]. Circ-SMAD7 restrained ESCC cell proliferation and migration to act as a tumor inhibitor [24]. Our results exhibited that hsa_circ_0000277 knockdown resulted in inhibition of proliferation, colony formation, and cell cycle progression in ESCC cells. Hsa_circ_0000277 was confirmed to facilitate cell malignant development of ESCC. DDP is a common chemotherapeutic agent in the treatment of ESCC [25]. Long ncRNA CCAT1 induced chemoresistance of ESCC cells to DDP by targeting the miR-143/PLK1/BUBR1 axis [18], and lncRNA LINC00337 promoted DDP resistance in ESCC cells through upregulating TPX2 by recruiting E2F4 [26]. The current data indicated that hsa_circ_0000277 silence decreased IC50 of DDP and accentuated the DDP-mediated cell apoptosis, which revealed that hsa_circ_0000277 elevated DDP chemoresistance in ESCC cells. However, the regulatory mechanism of hsa_circ_0000277 in cancer progression and chemoresistance needed further exploration.

The “miRNAs sponges” functions of circRNAs have been emerged in various kinds of cancers [27, 28]. For ESCC, Lan et al. reported that circRAD23B contributed to ESCC cell proliferation and invasion through the sponge effect on miR-5095 [29]. Liu et al. explained that silencing cZNF292 upregulated miR-206 to repress cell growth and metastasis in ESCC [30]. Circ_0001971 contributed to carcinogenesis and chemoresistance via sponging miR-194/miR-204 in oral squamous cell carcinoma [31]. Herein, miR-873-5p was identified as a target of hsa_circ_0000277. The rescued analysis demonstrated that hsa_circ_0000277 regulated ESCC cellular progression and DDP resistance by sponging miR-873-5p. In addition, SOX4 was regarded as a downstream gene with the negative regulation by miR-873-5p in ESCC cells. MiR-873-5p has been proved as a tumor suppressor in papillary thyroid cancer by targeting CXCL16 [32] and in colon cancer via the inhibition of TUSC3 [33]. By performing the reverted assays, we also found that miR-873-5p functioned as an tumor inhibitor and a DDP sensitizer in ESCC cells via downregulating SOX4. Due to the positive effect of hsa_circ_0000277 on SOX4 level by targeting miR-873-5p, we then concluded that the regulatory function of hsa_circ_0000277 in ESCC was partly achieved by the miR-873-5p/SOX4 axis. This signal axis was also confirmed in the regulation of tumor growth and chemosensitivity in vivo.

Wnt/β-catenin is a pivotal signaling pathway in regulating oncogenesis and chemoresistance of human cancers [34–36]. HPV-16 E6 was shown to induce cell growth in ESCC by activating the Wnt/β-catenin pathway [37] and SPINK5 acted as a tumor inhibitor in ESCC through blocking the Wnt/β-catenin pathway [38]. These evidences demonstrated that Wnt/β-catenin signal activation was associated with ESCC progression. SOX4 expression could control the Wnt/β-catenin pathway in many cancers, such as melanoma [39] and endometrial cancer [40]. Through the detection of related proteins, we verified that hsa_circ_0000277 knockdown inhibited the Wnt/β-catenin pathway via the regulation of miR-873-5p/SOX4 axis. Thus, hsa_circ_0000277 targeted miR-873-5p/SOX4 to regulated cell progression and DDP resistance in ESCC via the Wnt/β-catenin pathway.

## Conclusion

In general, hsa_circ_0000277 promoted the malignant progression and chemoresistance in ESCC via regulating miR-873-5p/SOX4-mediated Wnt/β-catenin signaling pathway. Hsa_circ_0000277/miR-873-5p/SOX4/Wnt/β-catenin was a novel molecular pathomechanism and resistance mechanism in ESCC.

## Supplementary Information

**Additional file 1: Supplementary Fig. 1.** The detection of apoptotic proteins. (A-H) The protein levels of cleaved-PARP and cleaved-caspase3 were detected by western blot for Fig. 3K-L (A-B), Fig. 5M-N (C-D), Fig. 7L-M (E-F) and Fig. 9 (G-H). *P < 0.05. **Supplementary Fig. 2.** Knockdown of hsa_circ_0000277 reduced proliferation and induced apoptosis in ESCC cells. (A-B) Cell proliferation by EdU assay (A) and apoptosis by flow cytometry (B) were performed after EC9706 and KYSE30 cells were transfected with sh-NC, sh-circ_0000277#1, sh-circ_0000277#2. *P < 0.05.

**Additional file 2.**

## Data Availability

The datasets used and/or analyzed during the current study are available from the corresponding author on reasonable request.

## Abbreviations

EC: Esophageal cancer
IC50: Inhibitory concentration
DDP: Cisplatin
RIP: RNA immunoprecipitation
miR-873-5p: microRNA-873-5p
SOX4: Sry-related high-mobility group box 4
