# Supplementary material for: Circular RNA hsa_circ_0000277 promotes tumor progression and DDP resistance in esophageal squamous cell carcinoma
Source: BMC Cancer. 2022 Mar 4;22:238. doi: 10.1186/s12885-022-09241-9 (PMC8895546; doi:10.1186/s12885-022-09241-9)

**Supplementary Fig. 1. The detection of apoptotic proteins.** (A-H) The protein levels of cleaved-PARP and cleaved-caspase3 were detected by western blot for Fig. 3K-L (A-B), Fig. 5M-N (C-D), Fig. 7L-M (E-F) and Fig. 9 (G-H). **P* < 0.05. **
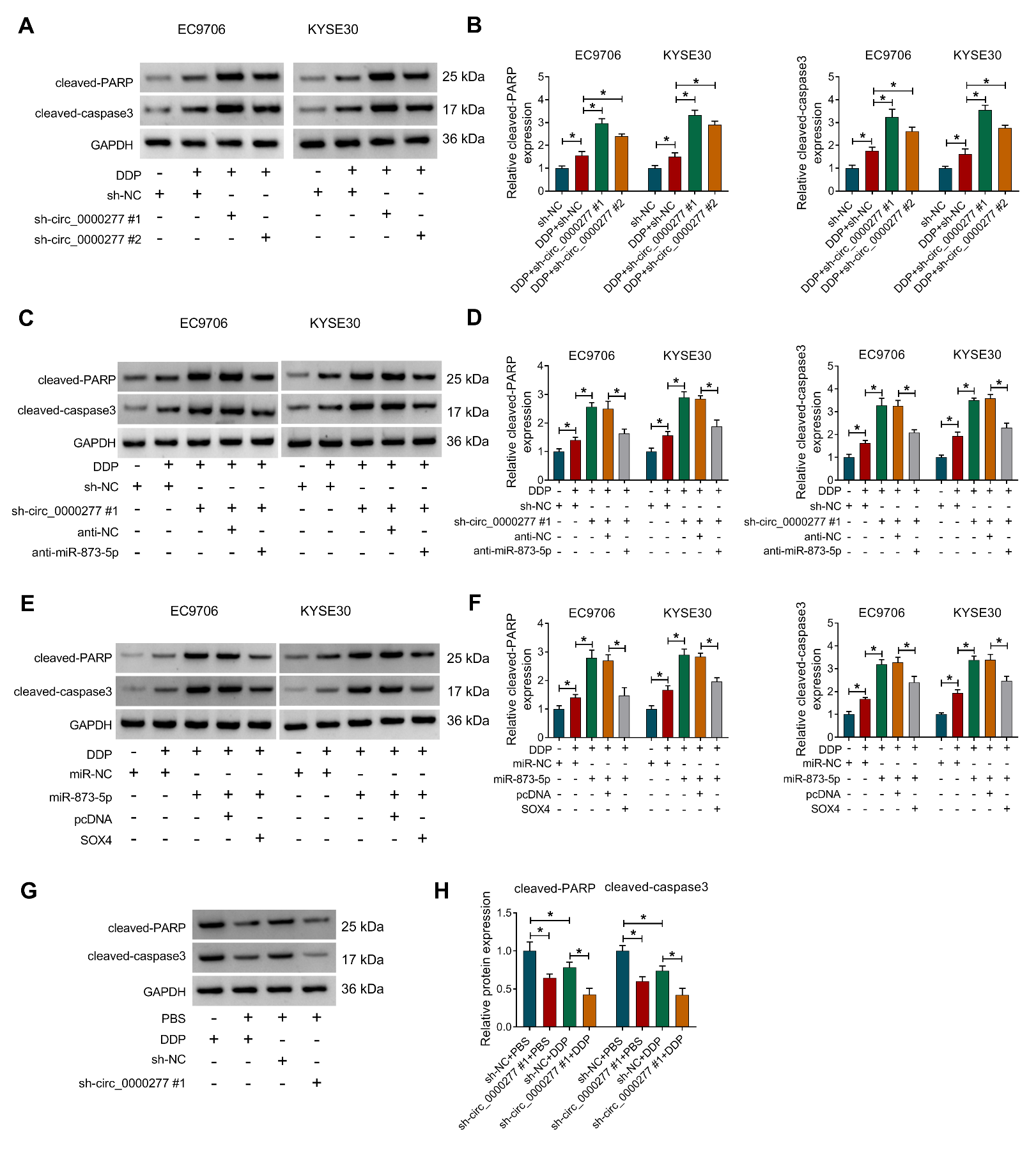
**

**Supplementary Fig. 2. Knockdown of hsa_circ_0000277 reduced proliferation and induced apoptosis in ESCC cells.** (A-B) Cell proliferation by EdU assay (A) and apoptosis by flow cytometry (B) were performed after EC9706 and KYSE30 cells were transfected with sh-NC, sh-circ_0000277#1, sh-circ_0000277#2. **P* < 0.05.
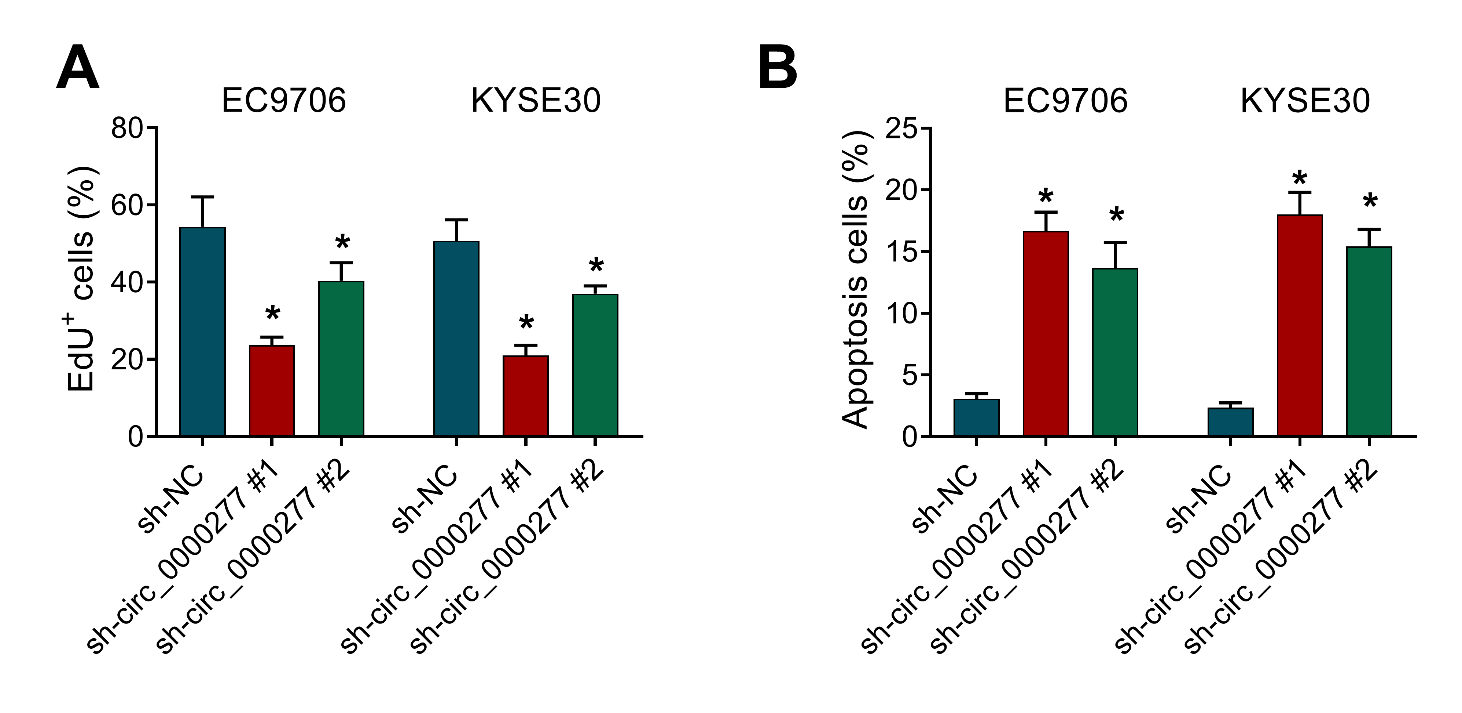

Supplement: Supplementary file 1 — Additional file 1: Supplementary Fig. 1. The detection of apoptotic proteins. (A-H) The protein levels of cleaved-PARP and cleaved-caspase3 were detected by western blot for Fig. 3K-L (A-B), Fig. 5M-N (C-D), Fig. 7L-M (E-F) and Fig. 9 (G-H). *P < 0.05. Supplementary Fig. 2. Knockdown of hsa_circ_0000277 reduced proliferation and induced apoptosis in ESCC cells. (A-B) Cell proliferation by EdU assay (A) and apoptosis by flow cytometry (B) were performed after EC9706 and KYSE30 cells were transfected with sh-NC, sh-circ_0000277#1, sh-circ_0000277#2. *P < 0.05. [file 12885_2022_9241_MOESM1_ESM.docx]
