# Supplementary material for: Circular RNA hsa_circ_0000277 promotes tumor progression and DDP resistance in esophageal squamous cell carcinoma
Source: BMC Cancer. 2022 Mar 4;22:238. doi: 10.1186/s12885-022-09241-9 (PMC8895546; doi:10.1186/s12885-022-09241-9)
Supplement: Supplementary file 2 — Additional file 2. [file 12885_2022_9241_MOESM2_ESM.pdf]

Fig6G

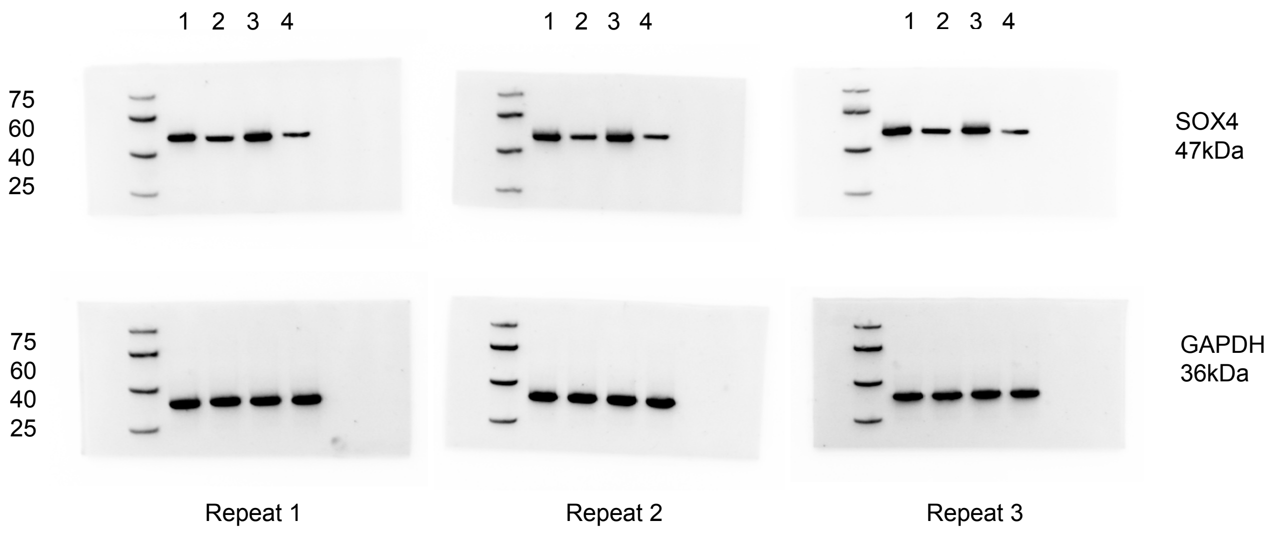

EC9706

KYSE30

1 miR-NC

2 miR-873-5p

3 miR-NC

4 miR-873-5p

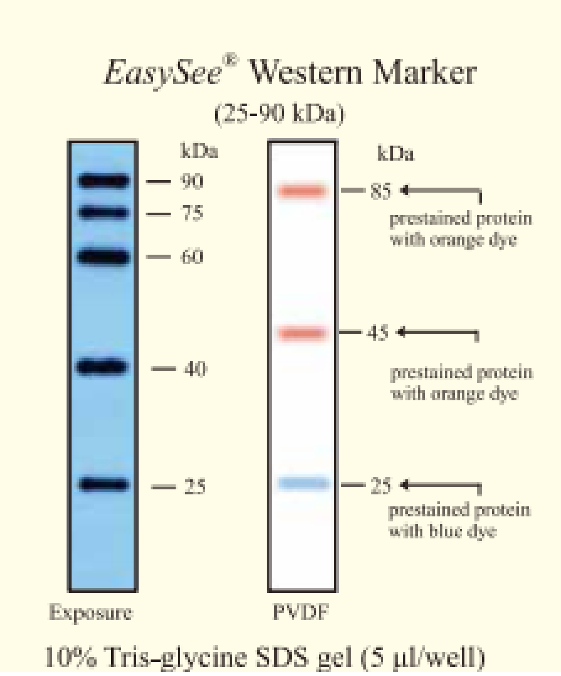

Fig7B

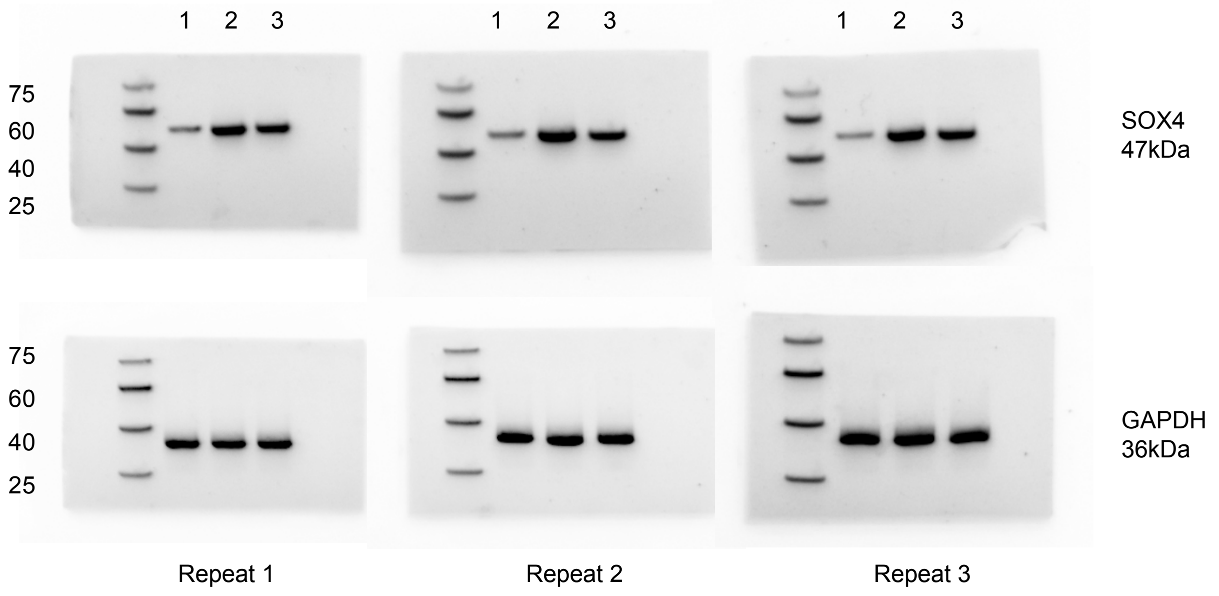

1 HET-1A 2 EC9706 3 KYSE30

Fig7C

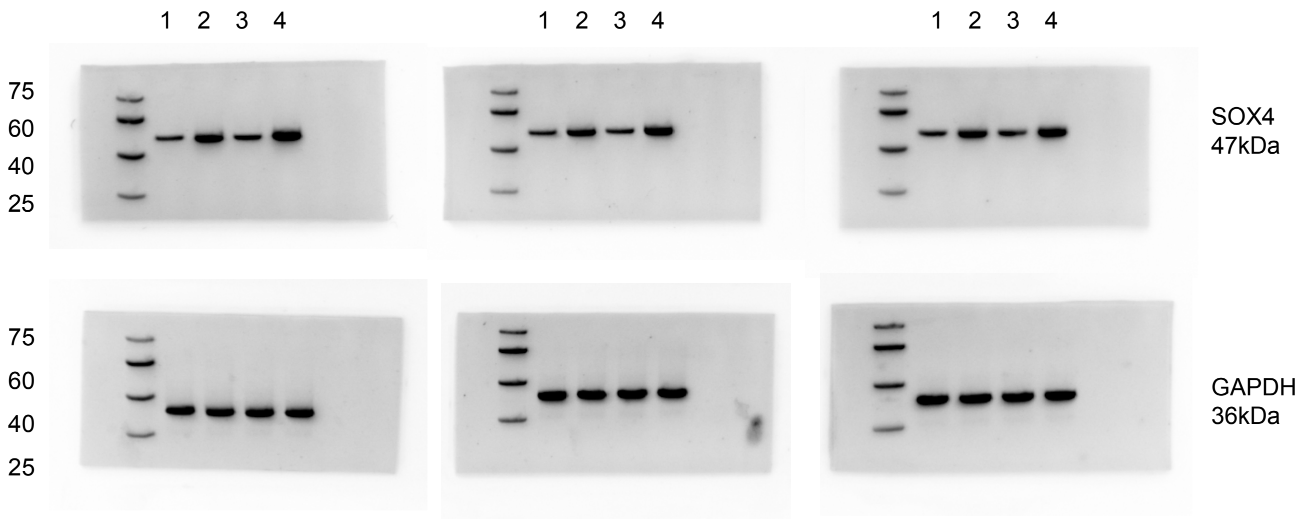

EC9706

KYSE30

1 pcDNA 2 SOX4 3 pcDNA 4 SOX4

Fig8C

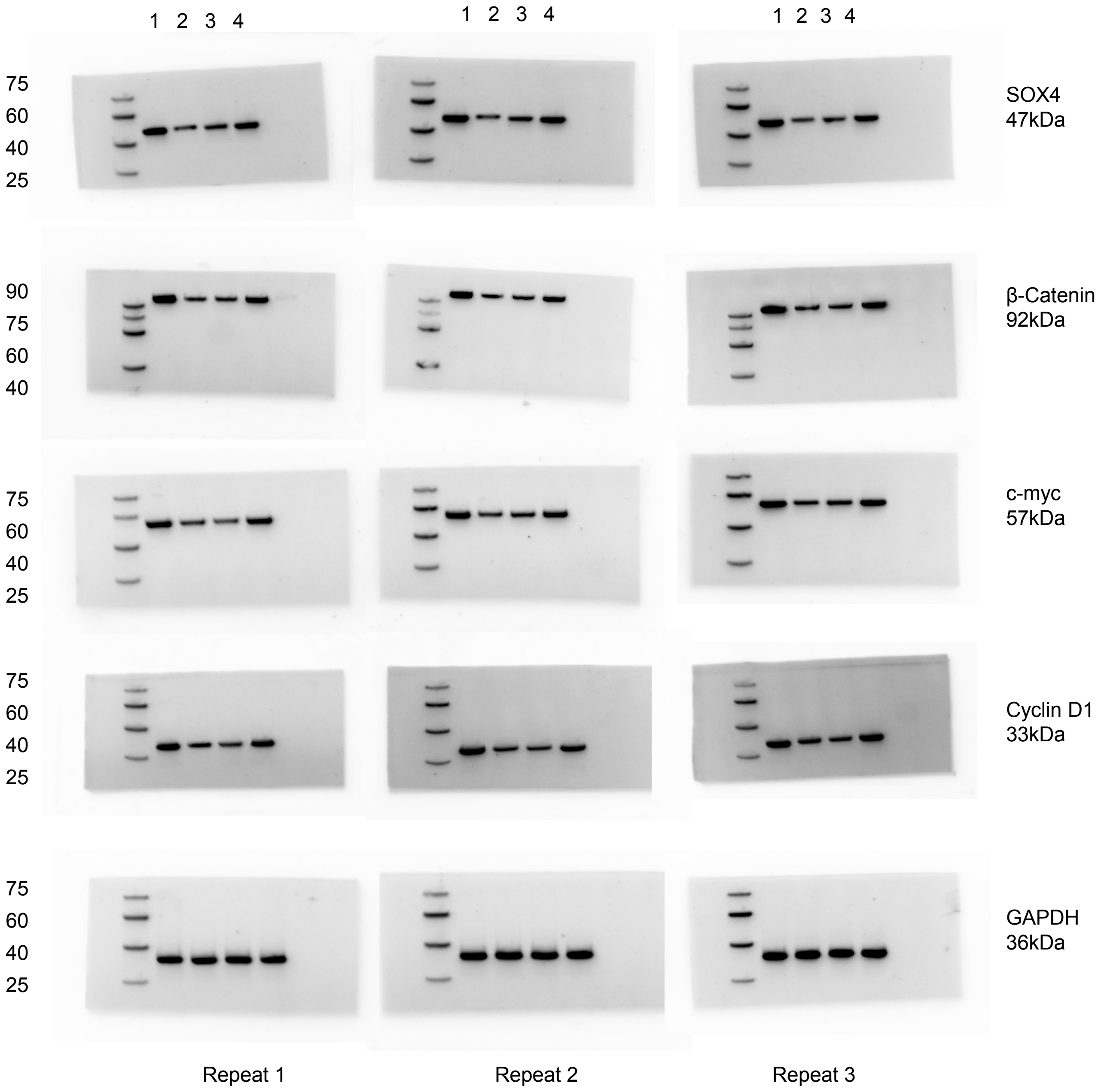

1 sh-NC 2 sh-has_circ_0000277 #1

3 sh-has_circ_0000277 #1+anti-NC

4 sh-has_circ_0000277 #1+anti-miR-873-5p

Fig8D

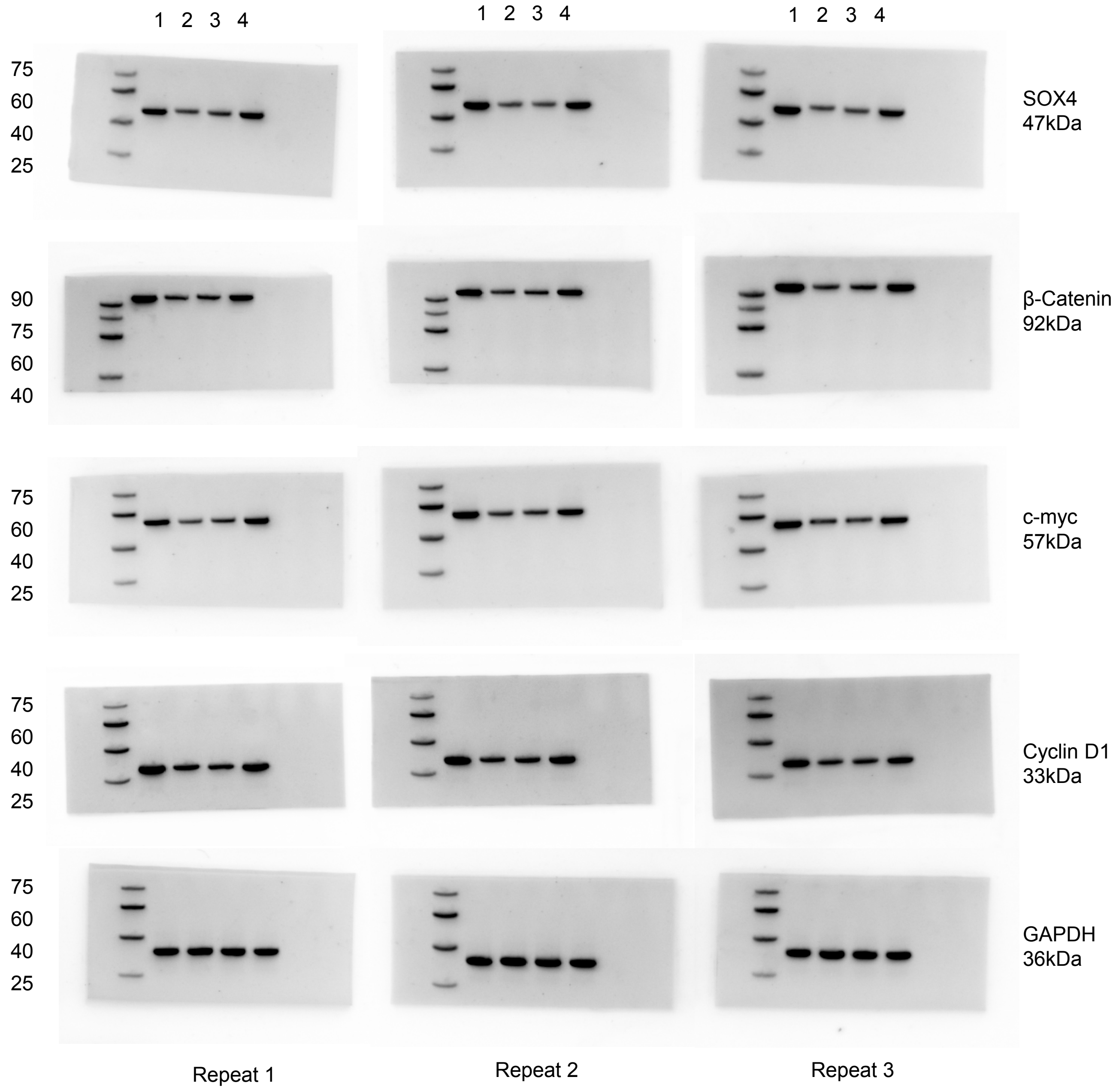

1 sh-NC 2 sh-has_circ_0000277 #1

3 sh-has_circ_0000277 #1+anti-NC

4 sh-has_circ_0000277 #1+anti-miR-873-5p

Fig9E

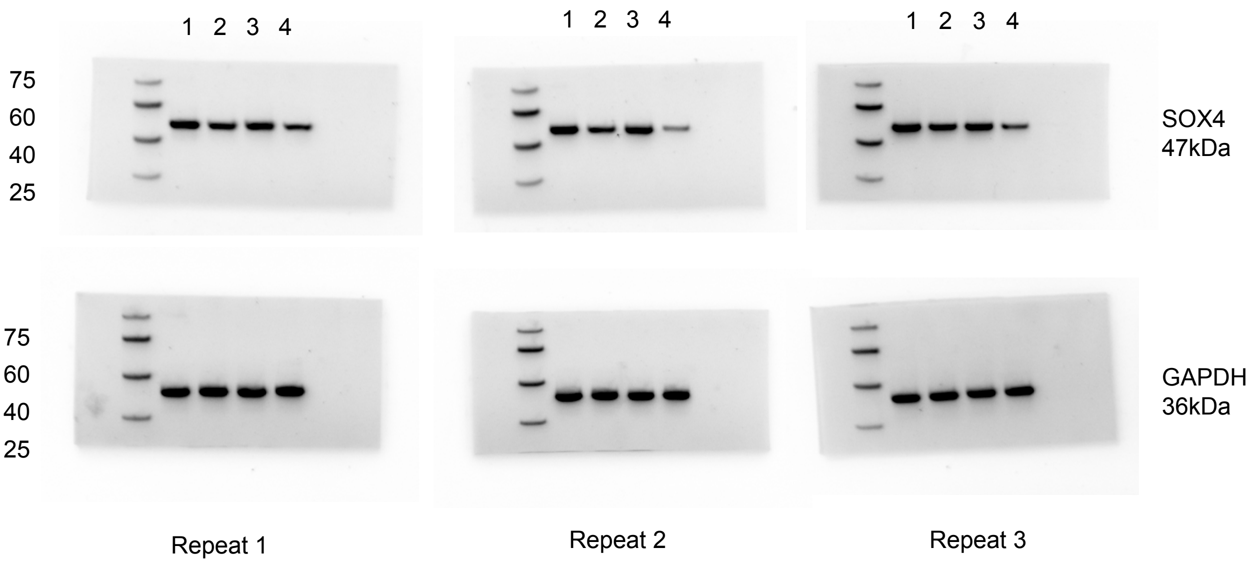

1 sh-NC+PBS 2 sh-has_circ_0000277 #1+PBS

3 sh-NC+DDP 4 sh-has_circ_0000277 #1+DDP

Supplementary Fig 1A   EC9706

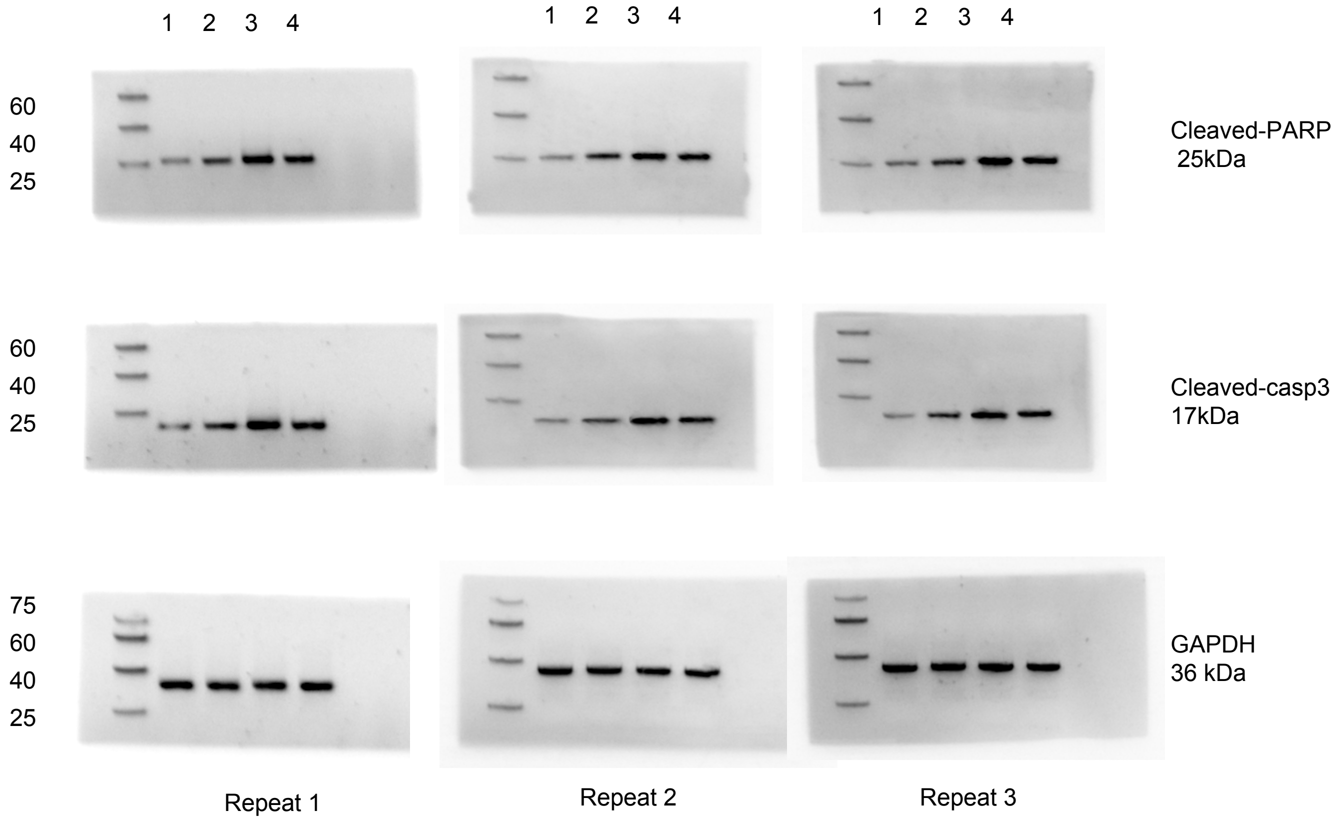

1 sh-NC   3 sh-has_circ_0000277 #1+DDP

2 sh-NC+DDP   4 sh-has_circ_0000277 #2+DDP

Supplementary Fig 1A

KYSE30

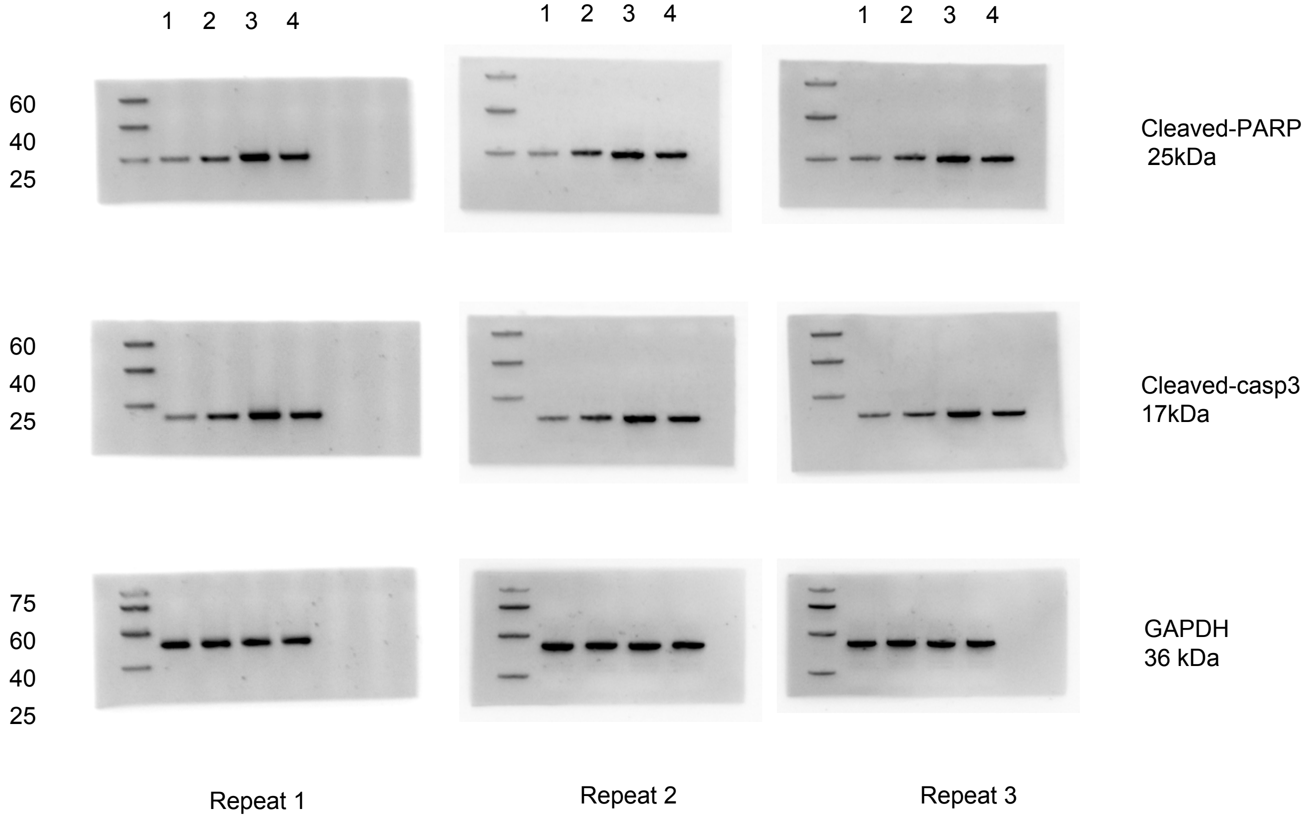

1 sh-NC 3 sh-has_circ_0000277 #1+DDP

2 sh-NC+DDP 4 sh-has_circ_0000277 #2+DDP

Supplementary Fig 1C EC9706

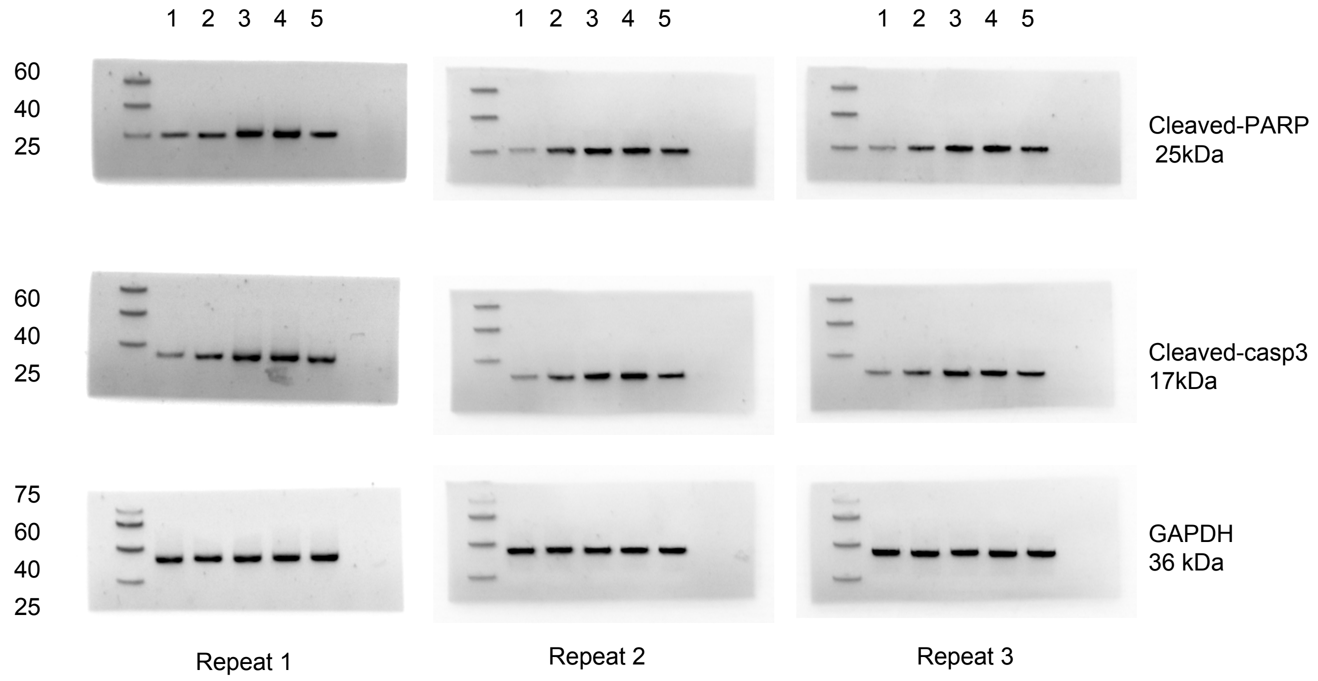

1 sh-NC

2 sh-NC+DDP

3 sh-has_circ_0000277 #1+DDP

4 sh-has_circ_0000277 #1+anti-NC+DDP

5 sh-has_circ_0000277 #1+anti-miR-873-5p+DDP

Supplementary Fig 1C KYSE30

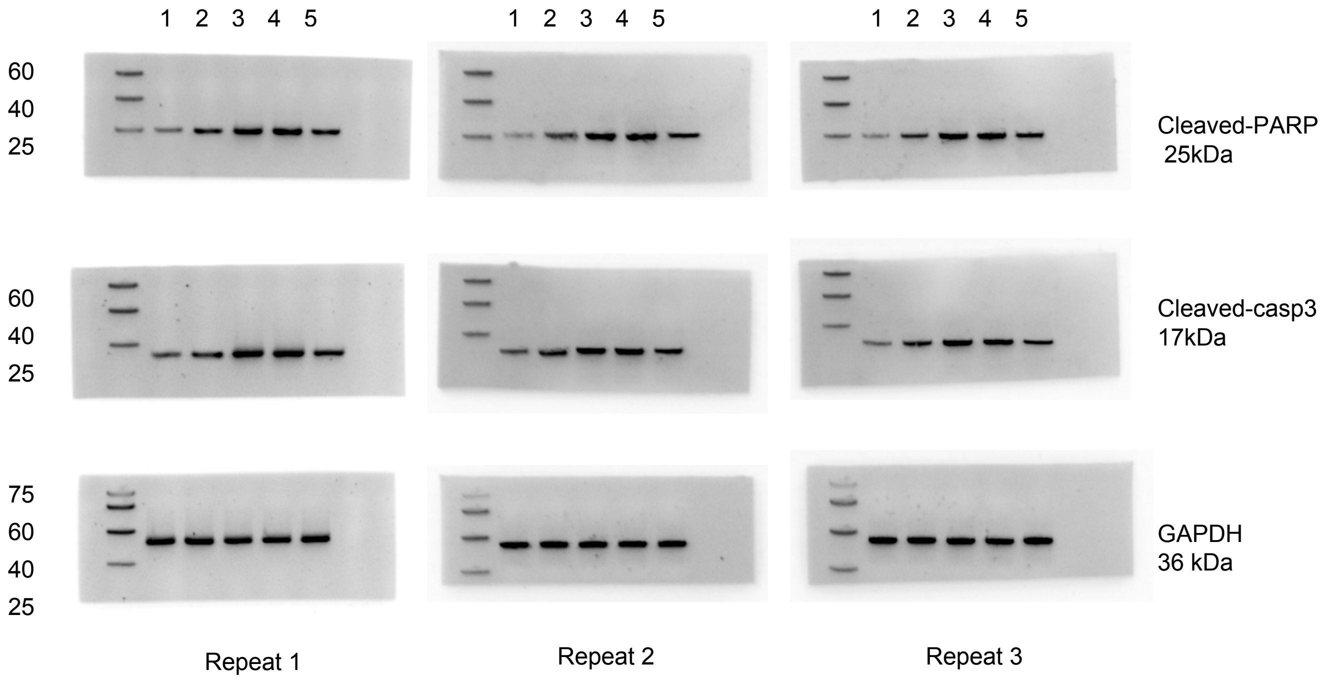

1 sh-NC

2 sh-NC+DDP

3 sh-has_circ_0000277 #1+DDP

4 sh-has_circ_0000277 #1+anti-NC+DDP

5 sh-has_circ_0000277 #1+anti-miR-873-5p+DDP

Supplementary Fig 1E EC9706

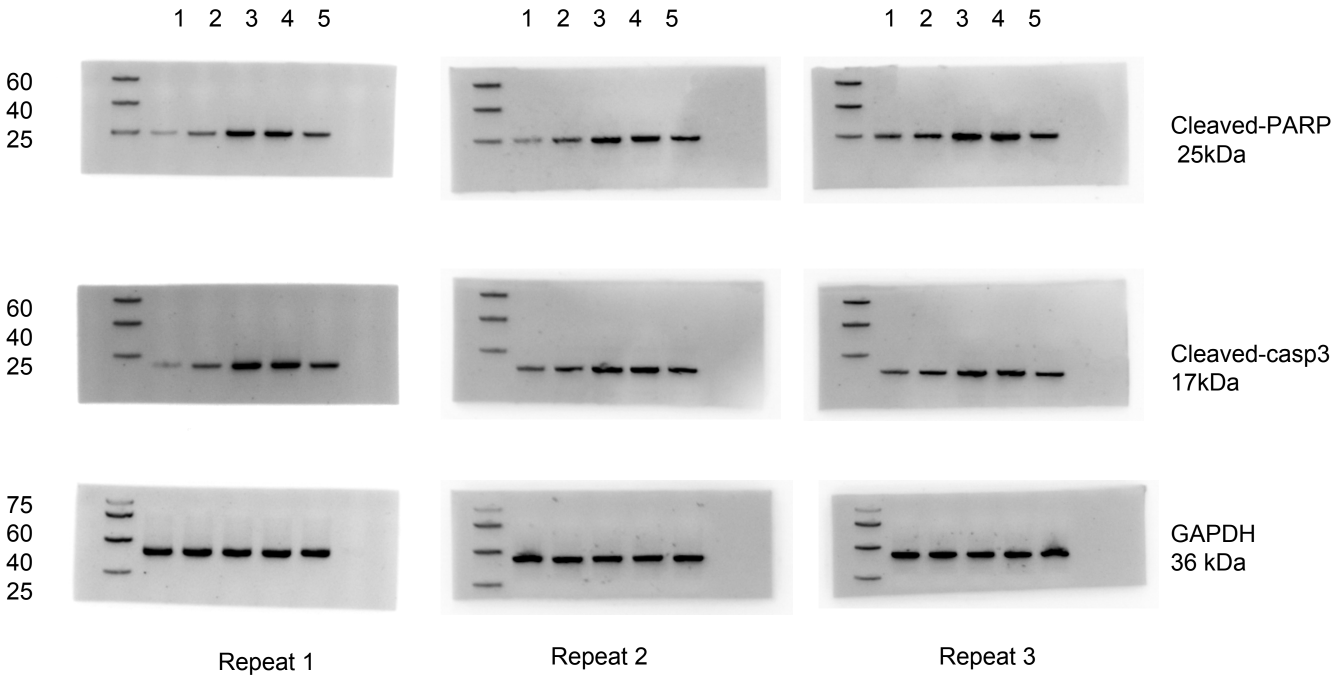

1 miR-NC

2 miR-NC+DDP

3 miR-873-5p+DDP

4 miR-873-5p+pcDNA+DDP

5 miR-873-5p+SOX4+DDP

Supplementary Fig 1E KYSE30

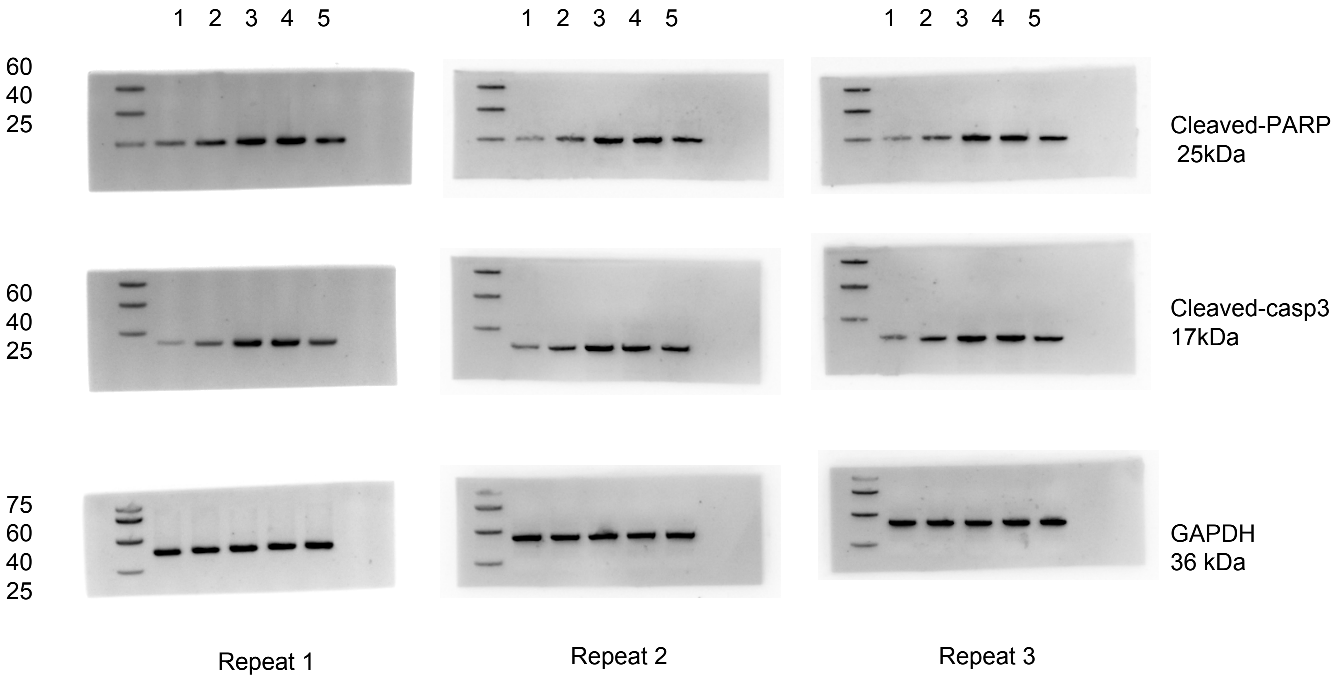

1 miR-NC

2 miR-NC+DDP

3 miR-873-5p+DDP

4 miR-873-5p+pcDNA+DDP

5 miR-873-5p+SOX4+DDP

Supplementary Fig 1G

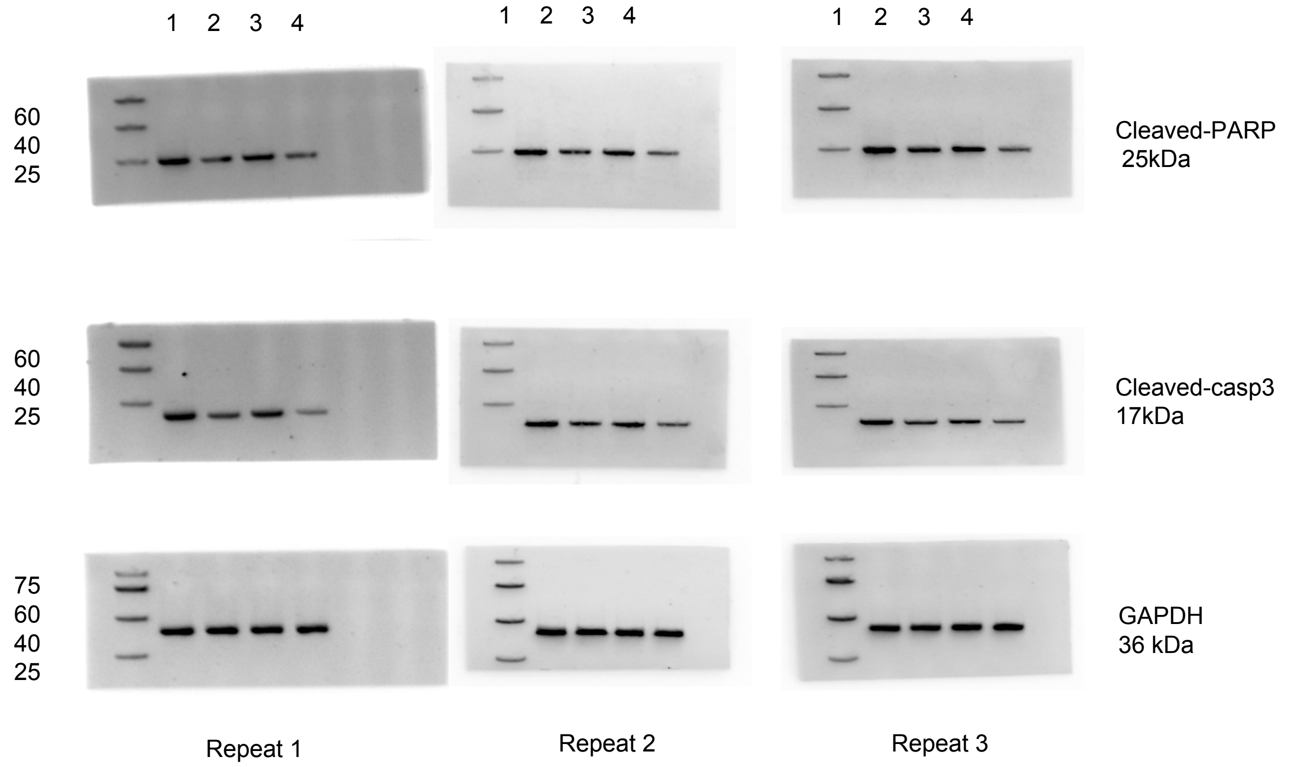

1 sh-NC+PBS

2 sh-has_circ_0000277 #1+PBS

3 sh-NC+DDP

4 sh-has_circ_0000277 #1+DDP
